# Biosynthesis of Silver Nanoparticles Using *Ocimum basilicum* Extract and Evaluation of Their Antifungal Efficacy Against Selected *Fusarium* Plant Pathogens

**DOI:** 10.3390/molecules31172985

**Published:** 2026-08-26

**Authors:** Ali O. E. Eltahir, Beauty E. Omoruyi, Mariska Lilly, Robert C. Luckay, Ahmed A. Hussein

**Affiliations:** 1 Chemistry Department, Cape Peninsula University of Technology, Symphony Rd., Bellville 7535, South Africa; aliomers250@gmail.com; 2 Department of Chemistry and Polymer Science, Stellenbosch University, Matieland, Stellenbosch 7602, South Africa; rcluckay@sun.ac.za; 3 Applied Microbial and Health Biotechnology Institute, Cape Peninsula University of Technology, Symphony Rd., Bellville 7535, South Africa; omoruyie@cput.ac.za (B.E.O.); lillym@cput.ac.za (M.L.)

**Keywords:** green synthesis, silver nanoparticles, fungi, antifungal, *Fusarium*, *Ocimum basilicum*

## Abstract

*Fusarium* species are significant phytopathogens responsible for major crop losses and contamination of food products with harmful mycotoxins, posing serious risks to food security and public health. In response to the limitations of conventional agrochemicals, this study explores sustainable antifungal strategies based on plant-derived products and nanotechnology. The aqueous extract of *Ocimum basilicum* was investigated as a bioresource for both direct antifungal activity and the green synthesis of silver nanoparticles (OB-AgNPs). Liquid chromatography–mass spectrometry (LC-MS) analysis of the *O. basilicum* aqueous extract revealed the presence of key compounds, including rosmarinic and chicoric acids, which can reduce and stabilize AgNPs. The biosynthesized nanoparticles were characterized using standard analytical techniques, including UV–visible spectroscopy (UV-Vis), Fourier-transform infrared spectroscopy (FTIR), dynamic light scattering (DLS), high-resolution transmission electron microscopy (HRTEM), and powder X-ray diffraction (PXRD). The OB-AgNPs exhibited a surface plasmon resonance (SPR) peak at 437 nm, a zeta potential of ~–16.3 mV, an average size of 28 nm (TEM) while the average hydrodynamic size is ≈57 nm (DLS). The synthesized AgNPs demonstrated stability in potato dextrose broth (PDB) for up to 24 h. The nanoparticles alongside the aqueous extract were evaluated against selected mycotoxigenic *Fusarium* species including *F. verticillioides*, *F. proliferatum*, *F. subglutinans*, *F. graminearum*, and *F. globosum*, following 96 h of exposure. Results showed that the aqueous *O. basilicum* extract exhibited limited antifungal activity, with significant inhibition observed only against *F. globosum* MRC 6122 at the highest concentration tested (400 ng/µL). In contrast, the biosynthesized OB-AgNPs demonstrated potent antifungal activity against most of the tested *Fusarium* strains, except for *F. proliferatum* MRC 8549 and MRC 8550, indicating substantially greater efficacy than the crude extract. These findings highlight the potential of *O. basilicum*-mediated AgNPs as effective and eco-friendly antifungal agents. The study underscores the value of integrating plant-based phytochemicals with nanotechnology for controlling *Fusarium* pathogens, while emphasizing the need for future studies to determine their effects on mycotoxin production, as well as their safety and phytotoxicity for agricultural applications.

## 1. Introduction

*Fusarium* species are major threats to global crop production due to their ability to produce mycotoxins, toxic secondary metabolites that compromise both plant health and food safety [1]. Key species such as *Fusarium verticillioides*, *F*. *graminearum* and *F*. *proliferatum* commonly contaminate cereals as well as fruits and vegetables, causing diseases including blights, ear and stalk rot, and crown and root rot, which result in substantial yield and quality losses [2]. These impacts extend beyond the field, contributing to food insecurity and nutritional decline during both pre- and post-harvest stages.

A critical concern is the accumulation of mycotoxins such as deoxynivalenol, nivalenol, zearalenone and fumonisins, particularly during storage. These toxins pose serious risks to human and animal health, rendering contaminated products unsafe for consumption [1]. Notably, *F. verticillioides* and *F. proliferatum* produce fumonisin B_1_ (FB_1_), classified by IARC as a Group 2B carcinogen and associated with oesophageal and liver cancers, neural tube defects and developmental disorders [3,4]. The prevalence and severity of *Fusarium* infections are strongly influenced by environmental factors, especially temperature, humidity and drought conditions, which shape their distribution across agroecological zones [5].

Silver nanoparticles (AgNPs) occupy a preeminent position in agricultural nanotechnology due to their exceptional physicochemical properties and high surface-to-volume ratio, which endows them with profound reactivity [6,7]. To optimize their field utility, current research emphasizes green or biogenic synthesis methods utilizing plant extracts and microorganisms [8]. This eco-friendly approach circumvents high energy demands and toxic chemical residues, leveraging natural biomolecules that act as sustainable reducing and capping agents to enhance nanoparticle stability and environmental biocompatibility [6,9]. A major frontier for these green-synthesized AgNPs is their powerful application in agricultural mycological control to safeguard crop yields [10]. Operating through multifaceted mechanisms including membrane disruption and targeted reactive oxygen species (ROS) induction, green AgNPs effectively combat destructive, multidrug-resistant phytopathogens [11]. Their dynamic interaction with microbial surfaces allows them to suppress fungal growth and inhibit spore germination without the ecotoxicological risks associated with conventional chemical fungicides [6,11]. Consequently, standardizing these biogenic formulations represents a critical step forward in establishing sustainable, target-specific antifungal strategies for modern crop protection [7,12].

Although agrochemicals are widely used to control plant pathogens, their environmental persistence and potential risks to food safety and public health remain significant concerns [13,14]. This has driven the search for safer, cost-effective and sustainable alternatives to manage fungal pathogens and reduce mycotoxin contamination. Silver nanoparticles have emerged as promising antimicrobial agents due to their enhanced physicochemical properties and broad-spectrum activity. Green synthesis approaches, particularly those using plant extract, offer eco-friendly and efficient routes for producing AgNPs with strong antimicrobial potential. These biosynthesized nanoparticles have demonstrated effectiveness against drug-resistant microbes and fungal pathogens, including *Fusarium* species, with potential applications in agriculture, food preservation and biomedicine [15,16,17].

Medicinal plants within the genus *Ocimum* (family Lamiaceae), notably *Ocimum basilicum*, possess a diverse array of bioactive compounds and are utilized extensively across various geographical regions in traditional medicine to treat a wide range of diseases. The extensive global cultivation of *O. basilicum* provides a highly sustainable and accessible source of biomass, with its leaves, flowering tops, stems, seeds, and essential oil serving as excellent precursors for extraction [18,19]. For green nanotechnology applications, this plant offers a rich reservoir of small-molecule reducing and stabilizing agents. Its non-volatile fraction is heavily concentrated with phenolic acids and flavonoids such as rosmarinic, caffeic, and chlorogenic acids, quercetin, and rutin which effectively drive the reduction of metal salts into nanoparticles [20]. Plant-derived extracts have been widely investigated as alternative disease-control agents, offering diverse mechanisms of action distinct from synthetic chemicals. Simultaneously, tannins, phenolic acids, flavonoids, proteins, amino acids and carbohydrates from natural sources serve as robust capping matrices that prevent nanoparticle agglomeration [21,22,23,24]. Beyond synthesis, these capping biomolecules pass on their inherent therapeutic traits to the resulting nanomaterials. The extract from *O. basilicum* contains similar secondary metabolites and possesses potent antioxidant [25,26], anti-inflammatory [27,28], antidiabetic [29,30] and antimicrobial [31,32,33] properties that actively disrupt pathogen membranes and inhibit multidrug-resistant biofilms [34]. This makes *O. basilicum* an ideal, dual-action agent for fabricating functionalized metallic nanoparticles with strong pre-clinical potential in anticancer, antimicrobial, antidiabetic, and targeted drug delivery systems [35,36,37].

The management of *Fusarium* diseases relies predominantly on synthetic fungicides, including benzimidazoles, triazoles, and strobilurins [38]. Although these fungicides can effectively reduce disease severity, their long-term use has led to the emergence of resistant fungal populations, reduced efficacy, environmental persistence, and concerns regarding non-target toxicity and chemical residues in food products [39]. Furthermore, conventional fungicides may not adequately prevent mycotoxin production, even when fungal growth is suppressed. These limitations highlight the urgent need for sustainable, environmentally friendly antifungal strategies that are effective against both fungal proliferation and mycotoxin-producing species [40].

Green-synthesized AgNPs using plant-derived compounds have emerged as promising alternatives because they combine the antimicrobial properties of phytochemicals with the enhanced bioactivity of nanomaterials [41]. Their multiple mechanisms of action reduce the likelihood of resistance development while offering a more sustainable approach to plant disease management. Although numerous studies have reported the green synthesis of AgNPs using plant extracts, relatively few have systematically evaluated whether nanoparticle synthesis enhances the antifungal efficacy of the corresponding crude extract against agriculturally important mycotoxigenic *Fusarium* species [42,43,44,45]. Furthermore, studies integrating phytochemical profiling, comprehensive nanoparticle characterization, and mechanistic biological evaluation remain limited. To address this gap, this study aimed to green-synthesize AgNPs using an aqueous extract of *O. basilicum* and systematically evaluate their antifungal efficacy alongside the crude extract against ten mycotoxigenic isolates from five *Fusarium* species. Although plant-mediated AgNPs, including those synthesized from *Ocimum* species, possess demonstrated antifungal properties, their application is often constrained by concentration-dependent effects and potential phytotoxicity at elevated doses [42,43,44,45]. Furthermore, direct comparisons between crude plant extracts and their corresponding biogenic AgNPs remain limited, particularly against *Fusarium* spp. Therefore, the biosynthesized AgNPs were comprehensively characterized using LC-MS, UV–Vis, FTIR, DLS, HRTEM, and PXRD, while the comparative antifungal activity was quantified using ATP-based viability assays and visualized via scanning electron microscopy (SEM).

## 2. Results

### 2.1. Chemical Profile of the Aqueous Extract

**Figure 1:**
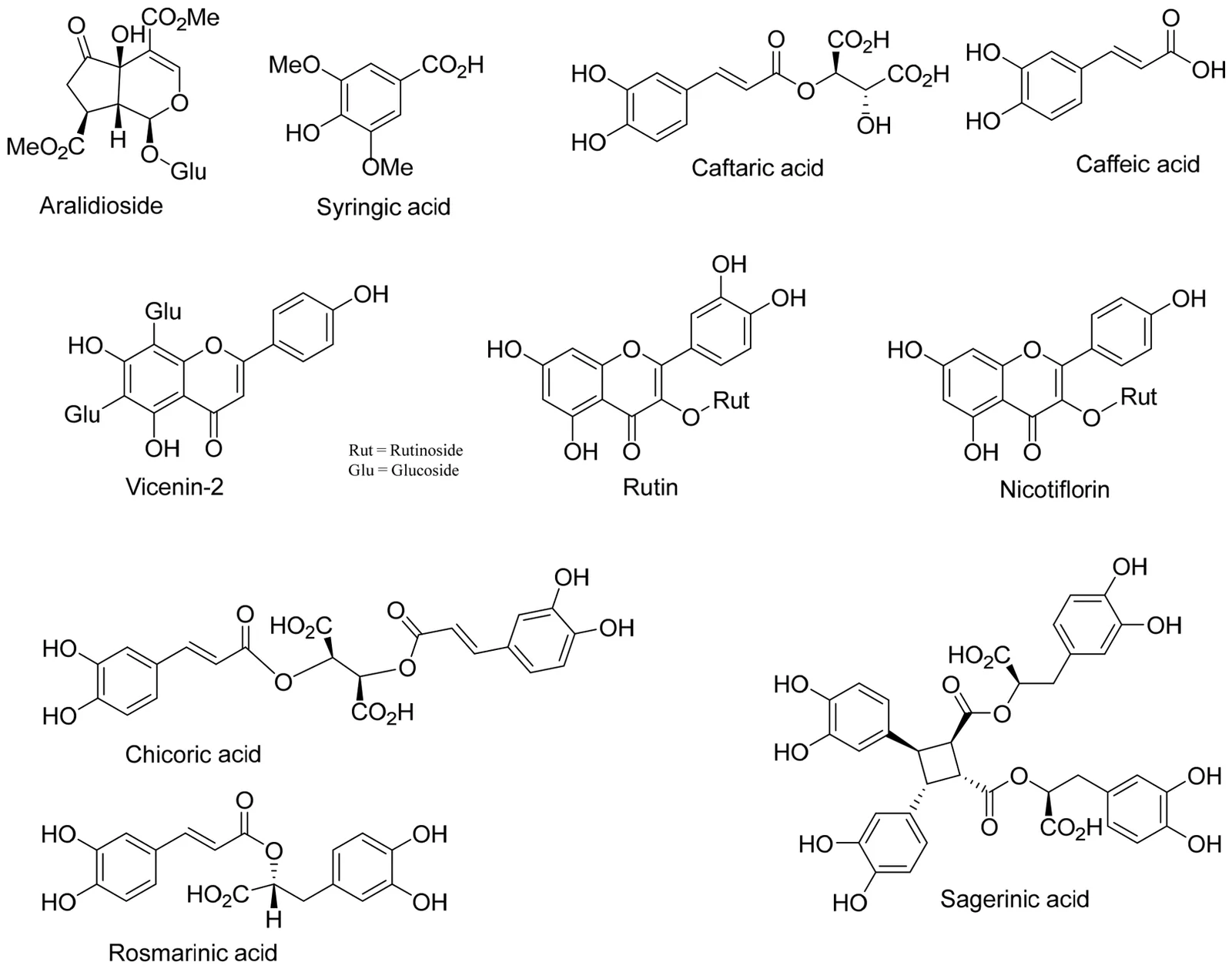
Chemical structures of the annotated phytochemicals from the *Ocimum basilicum* aqueous extract.

The aqueous extract of *O. basilicum* was analyzed by LC–MS, and the chromatogram (Figure S2) revealed ten major peaks at retention times of 5.662 (syringic acid); 6.132 (caftaric acid); 6.224 (aralidioside); 7.236 (caffeic acid); 7.355 (vicenin-2); 8.145 (chicoric acid); 8.237 (rutin); 8.611 (sagerinic acid); 8.681 (nicotiflorin); and 9.075 (rosmarinic acid) in ESI negative ionization mode. Rosmarinic acid showed the highest concentration (65.0 μg/mg TE) followed by vicenin-2 (42.0 μg/mg TE) and chicoric acid (39.6 μg/mg TE) (Figure 1 and Figure S2, Table S1).

### 2.2. Synthesis and Characterization of the Biosynthesized Silver Nanoparticles

**Figure 2:**
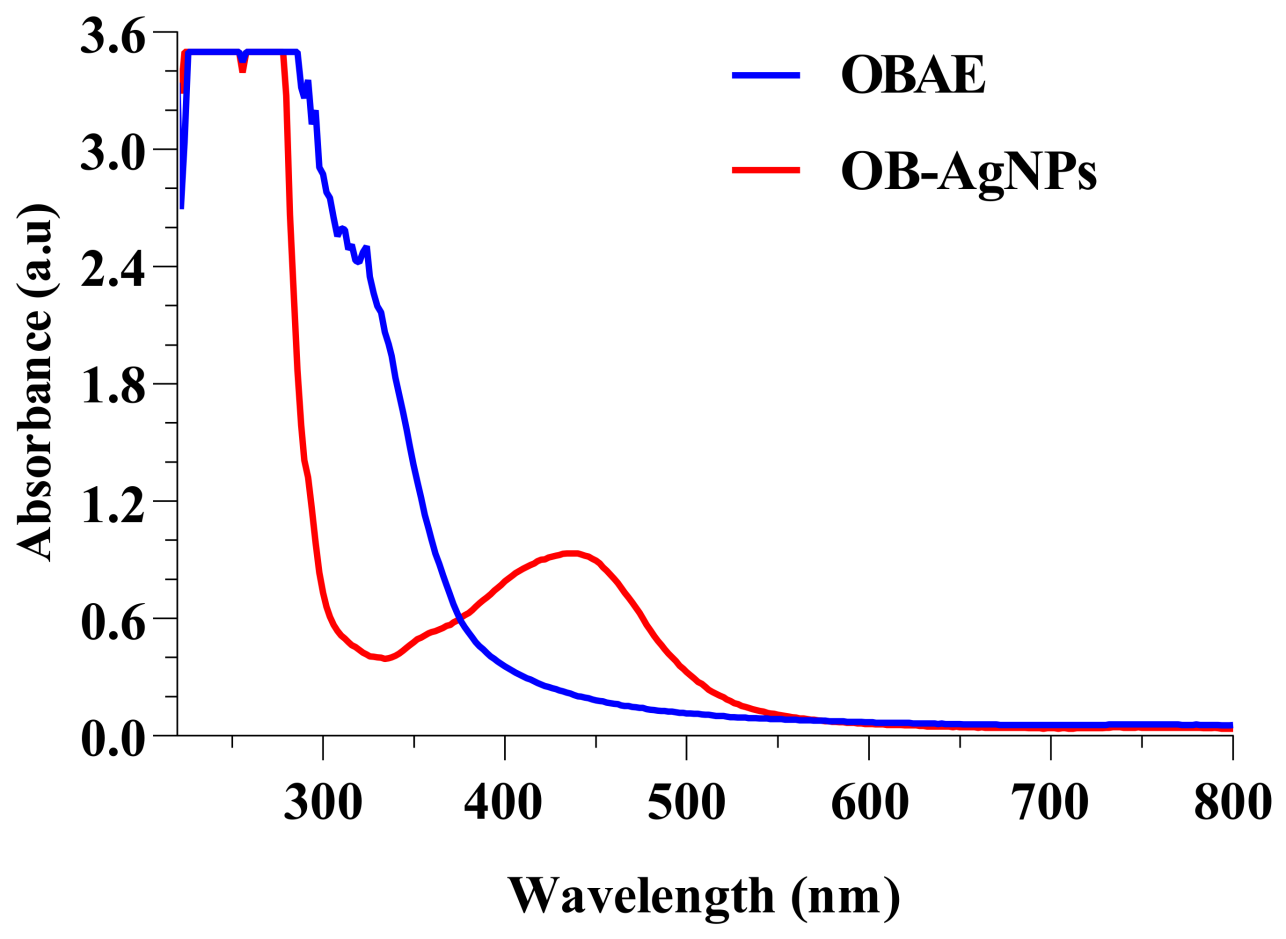
Ultraviolet–visible (UV-Vis) absorption spectra of *Ocimum basilicum* aqueous extract (OBAE) and the biosynthesized silver nanoparticles (OB-AgNPs).

Upon adding 10 mL of the aqueous extract (2 mg/mL) to 15 mL of 0.30 mM AgNO_3_ and heating at 70 °C for 1.0 h, the solution turned brown. This color change indicated the reduction of Ag^+^ to Ag^0^ and the formation of AgNPs (Figure 2 and Figure S1). The same ratio was used to scale up to 100 mL volume.

**Figure 3:**
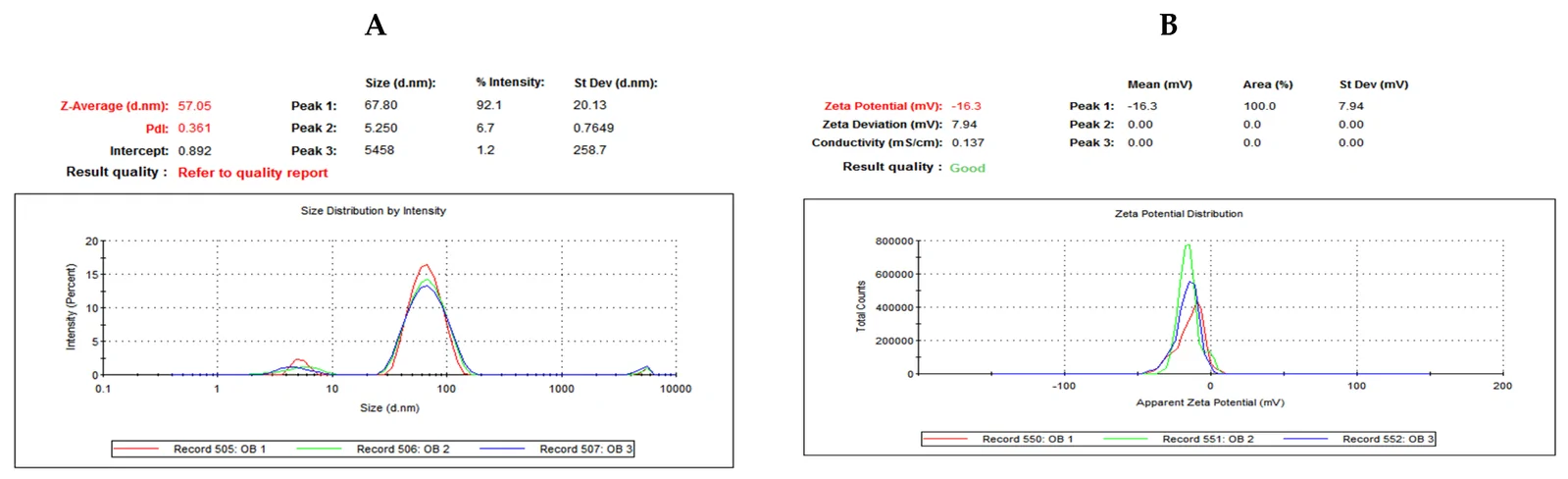
Zeta potential distribution (**A**) and hydrodynamic size distribution (**B**) of the biosynthesized silver nanoparticles (OB-AgNPs).

The UV-Vis spectrophotometric analysis of the biosynthesized silver nanoparticles using *O. basilicum* extract (OB-AgNPs) exhibited a maximum absorption peak λ_max_ at 437 nm (Figure 2), which is characteristic of the surface plasmon resonance (SPR) of AgNPs. Dynamic light scattering analysis revealed an average hydrodynamic size of 57.08 nm, with a polydispersity index (PDI) of 0.361, indicating a moderate disperse particle distribution. The surface charge and stability of the nanoparticles were evaluated *via* zeta potential (ZP) measurements, yielding a value of –16.3 mV, which suggests a stable colloidal suspension stabilized by negative surface charges (Figure 3).

Transmission electron microscopy (TEM) imaging confirmed that the actual core size of the nanoparticles ranged between 18.5 and 37.4 nm. This structural finding was further corroborated by X-ray diffraction (XRD) analysis; using the Debye–Scherrer equation, the average crystalline size of the OB-AgNPs was calculated to be ≈11.18 nm. The larger hydrodynamic diameter (57.08 nm) compared to the TEM and XRD core sizes is attributed to the hydration layer and capping biomolecules from the *O. basilicum* extract surrounding the nanoparticles (Table 1 and Figures S3 and S4).

**Figure 4:**
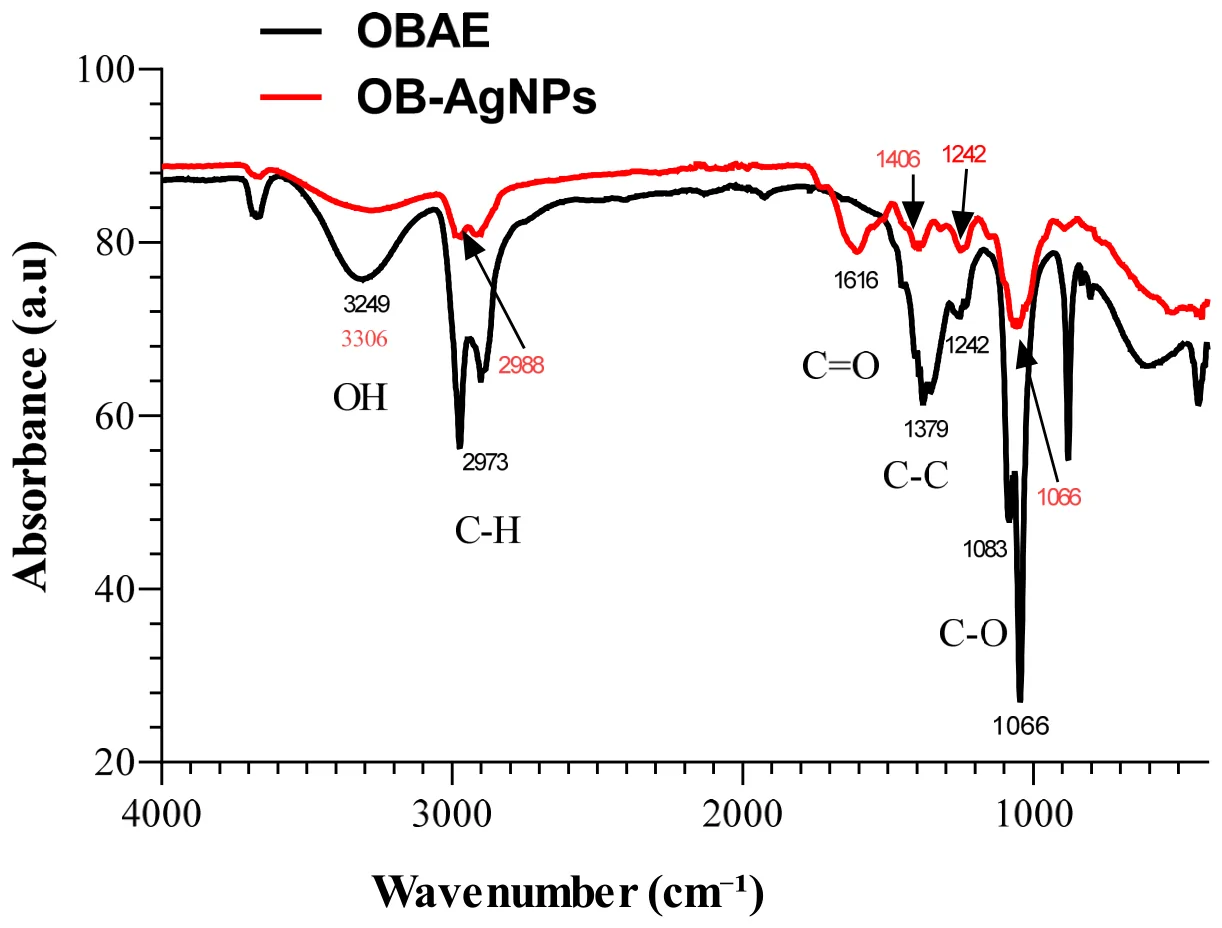
FTIR spectra of *Ocimum basilicum* aqueous extract (OBAE) and the biosynthesized silver nanoparticles (OB-AgNPs).

*Ocimum basilicum* contains numerous polyphenols, as mentioned above, with rosmarinic acid as a major constituent; these compounds possess phenolic hydroxyl (-OH) and carboxylic groups. A comparison of the FTIR spectra of the *O. basilicum* extract and OB-AgNPs (Figure 4) showed that the major peaks located at 1050–1076, 1600–1700, and 2900–3340 ${\mathrm{cm}}^{-1}$ corresponded to C–O (phenolic), C=O (α,β-unsaturated ketone, ester, and carboxylic groups), C–H (aromatics), and broad O–H stretching vibrations, respectively [20,46].

According to the IR spectra, the key functional groups present in *O. basilicum* were also preserved in the AgNPs. Notably, the C=O band at 1616 ${\mathrm{cm}}^{-1}$ appeared prominently in the synthesized NPs but was not observed in the intact extract. The spectra also revealed shifts in peak intensities and positions for the NPs compared to the intact extract. These spectroscopic changes before and after synthesis indicate the direct involvement of these functional groups in the reduction and capping processes.

### 2.3. Stability

**Figure 5:**
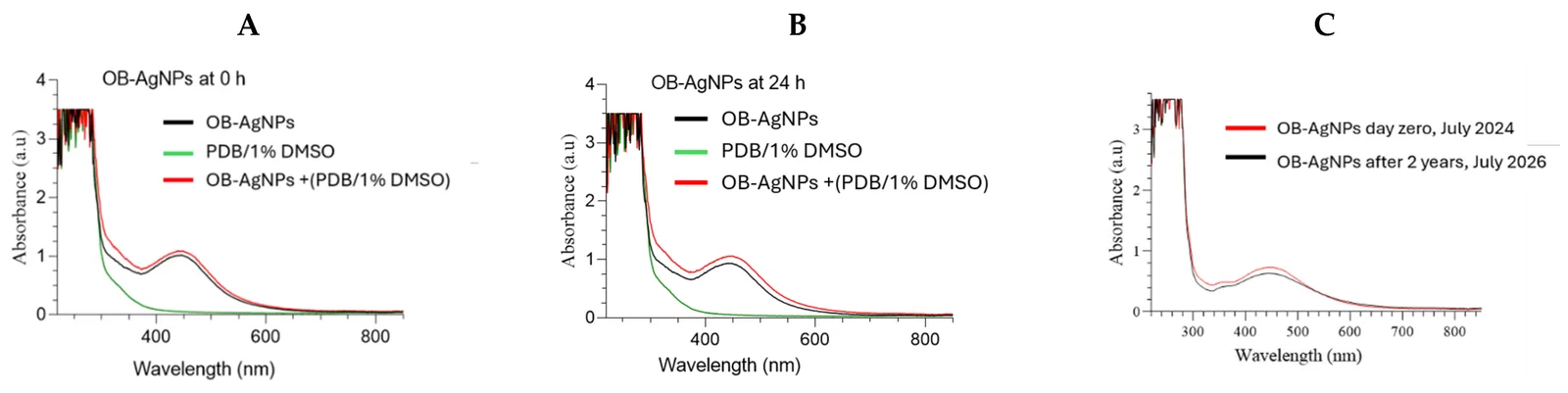
(**A**,**B**). Stability of the biosynthesized silver nanoparticles (OB-AgNPs) in potato dextrose broth (PDB) solution supplemented with 1% DMSO over a 24 h period. (**C**). Comparison of the initial (time-zero) and two-year post-synthesis ultraviolet–visible absorption spectra of the biosynthesized silver nanoparticles (OB-AgNPs).

To evaluate the stability of the synthesized AgNPs under experimental conditions, the particles were incubated overnight in potato dextrose broth (PDB) supplemented with 1% DMSO; this specific media matrix was selected as identical to the one utilized for the subsequent biological activity assays. UV-Vis spectrophotometric measurements were recorded at 0 and 24 h to monitor any structural or colloidal transitions. As illustrated in Figure 5A,B, the OB-AgNPs exhibited excellent stability over the 24 h testing period, maintaining a consistent surface plasmon resonance λ_max_ peak at ~437 nm after 24 h. Additionally, the stability of a sample stored at room temperature (20–30 °C) for two years was evaluated, revealing nearly identical measurements to those of the freshly prepared sample (Figure 5C and Figure S5).

### 2.4. Effect of Ocimum basilicum Extract and the Biosynthesized Silver Nanoparticles Against Fusarium Growth

**Figure 6:**
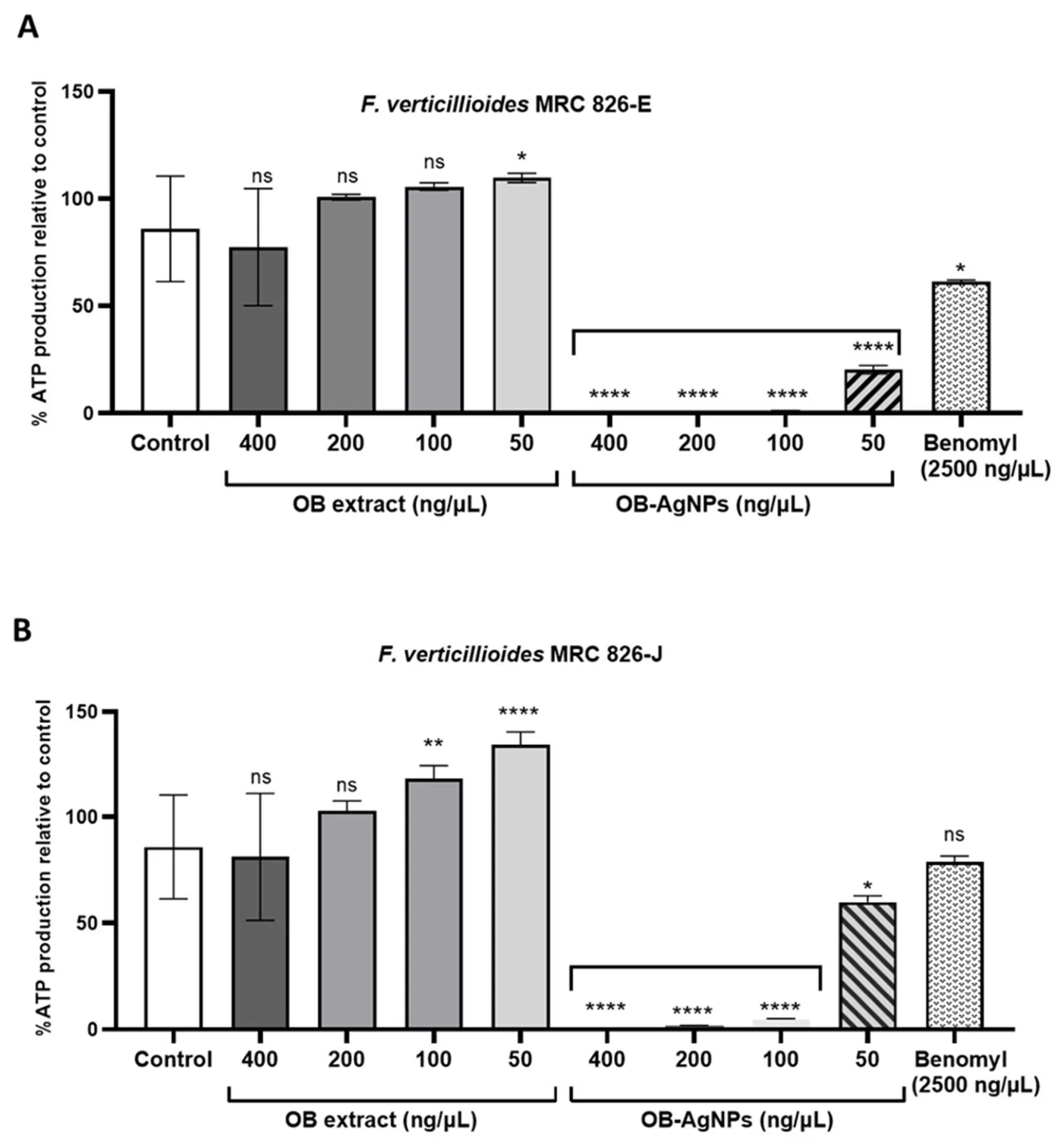

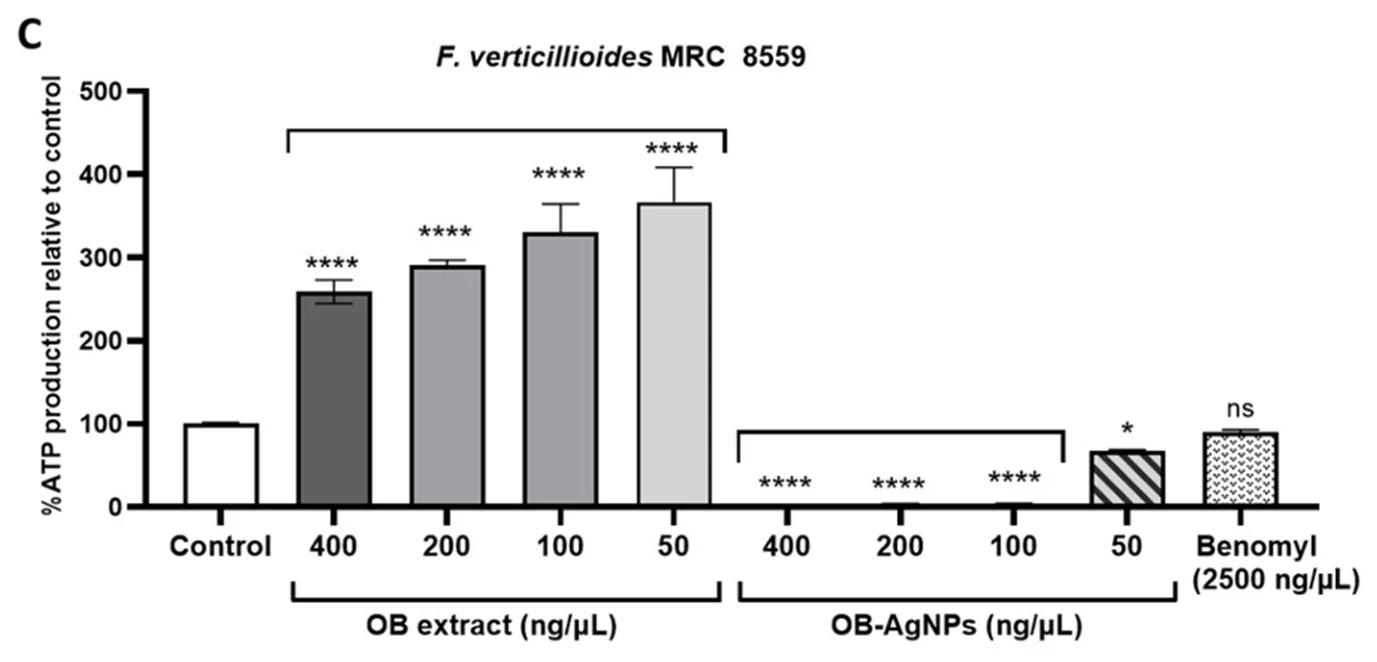
Conidial viability [percent adenosine triphosphate (ATP) production relative to unexposed control] of *Fusarium verticillioides* (**A**) MRC 826-E, (**B**) MRC 826-J, and (**C**) MRC 8559 after 96 h of exposure to *Ocimum basilicum* extract (OB extract) and the biosynthesized silver nanoparticles (OB-AgNPs). Data are presented relative to the unexposed control. ns, not statistically significant (*p* > 0.05); * are statistically significant at *p* < 0.05, ** *p* < 0.01, and **** *p* < 0.0001.

**Figure 7:**
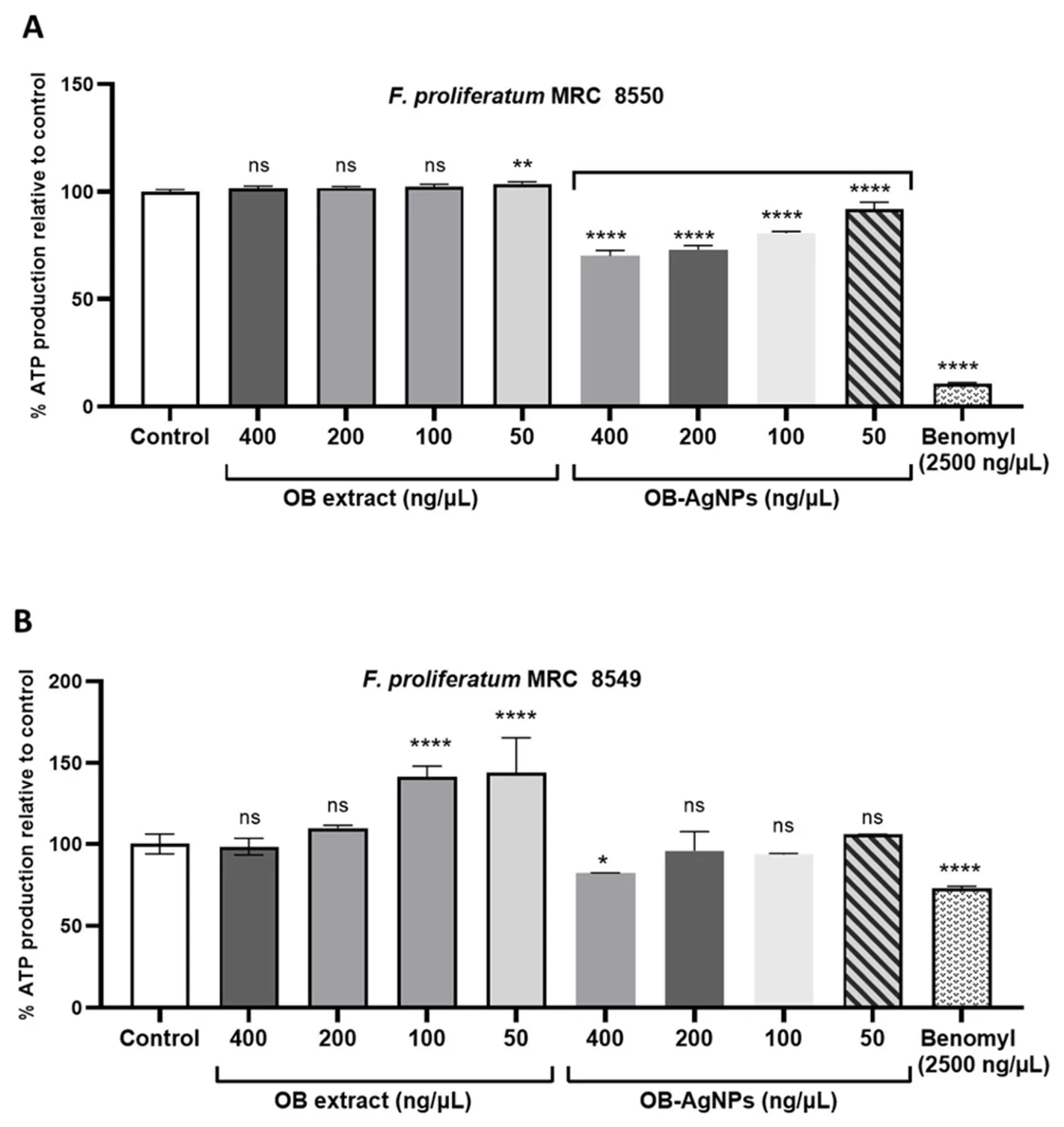
Conidial viability [percent adenosine triphosphate (ATP) production relative to unexposed control] of *Fusarium proliferatum* (**A**) MRC 8550 and (**B**) MRC 8549 after 96 h exposure to *Ocimum basilicum* extract (OB extract) and the biosynthesized silver nanoparticles (OB-AgNPs). Data are presented relative to the unexposed control. ns, not statistically significant (*p* > 0.05); * are statistically significant at *p* < 0.05, ** *p* < 0.01, and **** at *p* < 0.0001.

**Figure 8:**
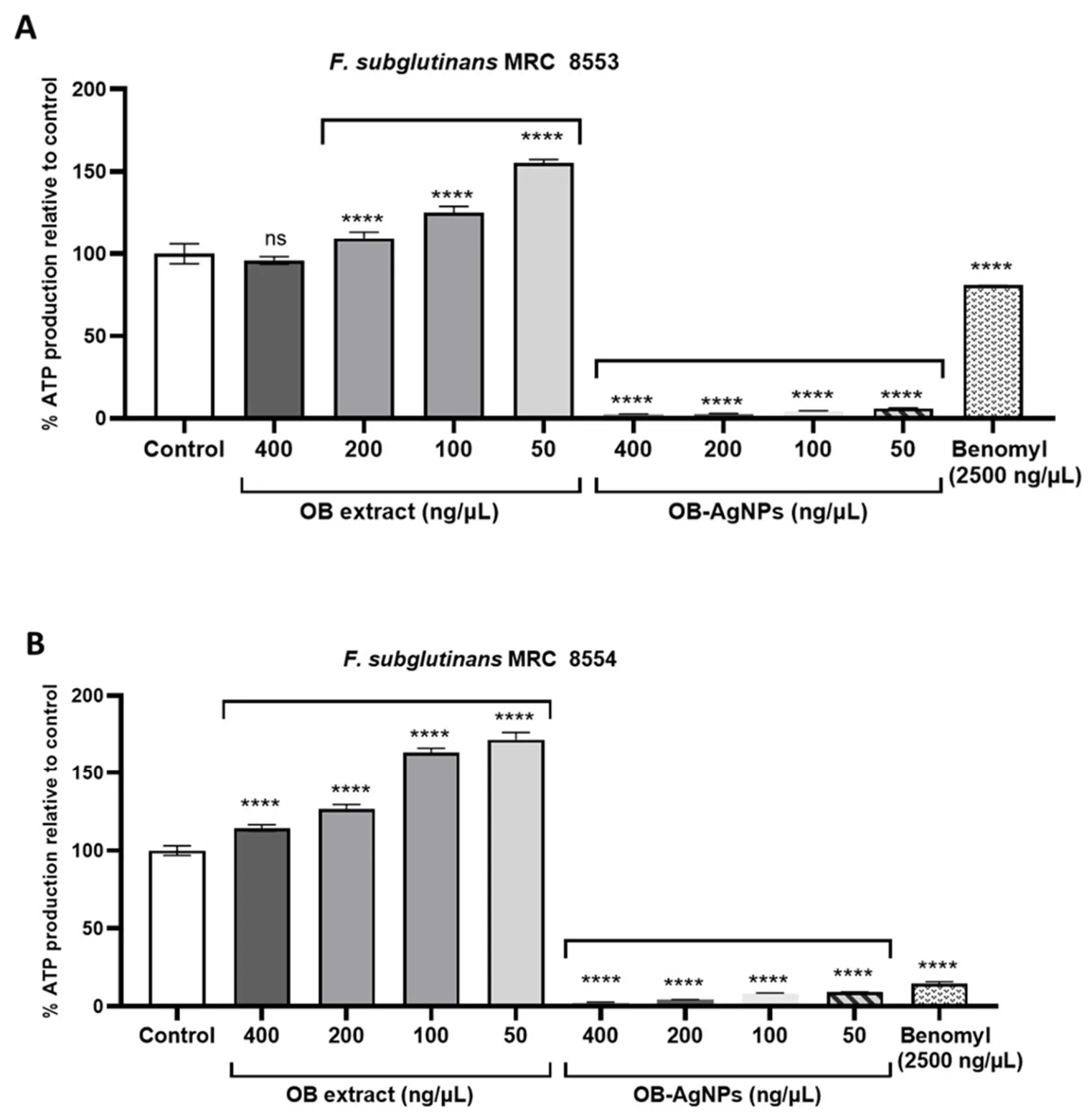
Conidial viability [percent adenosine triphosphate (ATP) production relative to unexposed control] of *Fusarium subglutinans* (**A**) MRC 8553 and (**B**) MRC 8554 after 96 h exposure to *Ocimum basilicum* extract (OB extract) and the biosynthesized silver nanoparticles (OB-AgNPs). Data are presented relative to the unexposed control. ns, not statistically significant (*p* > 0.05); **** are statistically significant at *p* < 0.0001.

**Figure 9:**
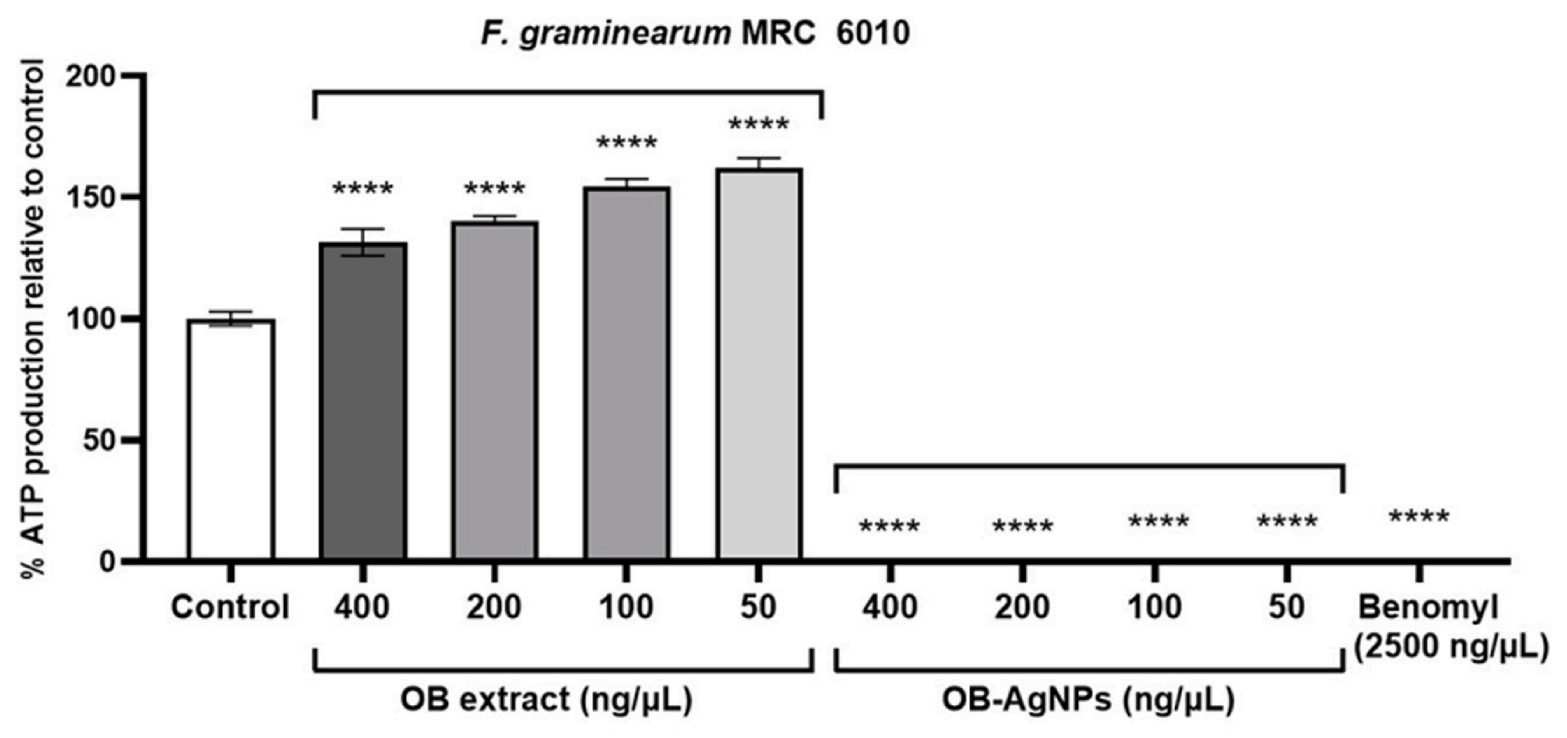
Conidial viability [percent adenosine triphosphate (ATP) production relative to unexposed control] of *Fusarium graminearum* MRC 6010 after 96 h exposure to *Ocimum basilicum* extract (OB extract) and the biosynthesized silver nanoparticles (OB-AgNPs). Data are presented relative to the unexposed control. Statistical differences are indicated as **** *p* < 0.0001.

**Figure 10:**
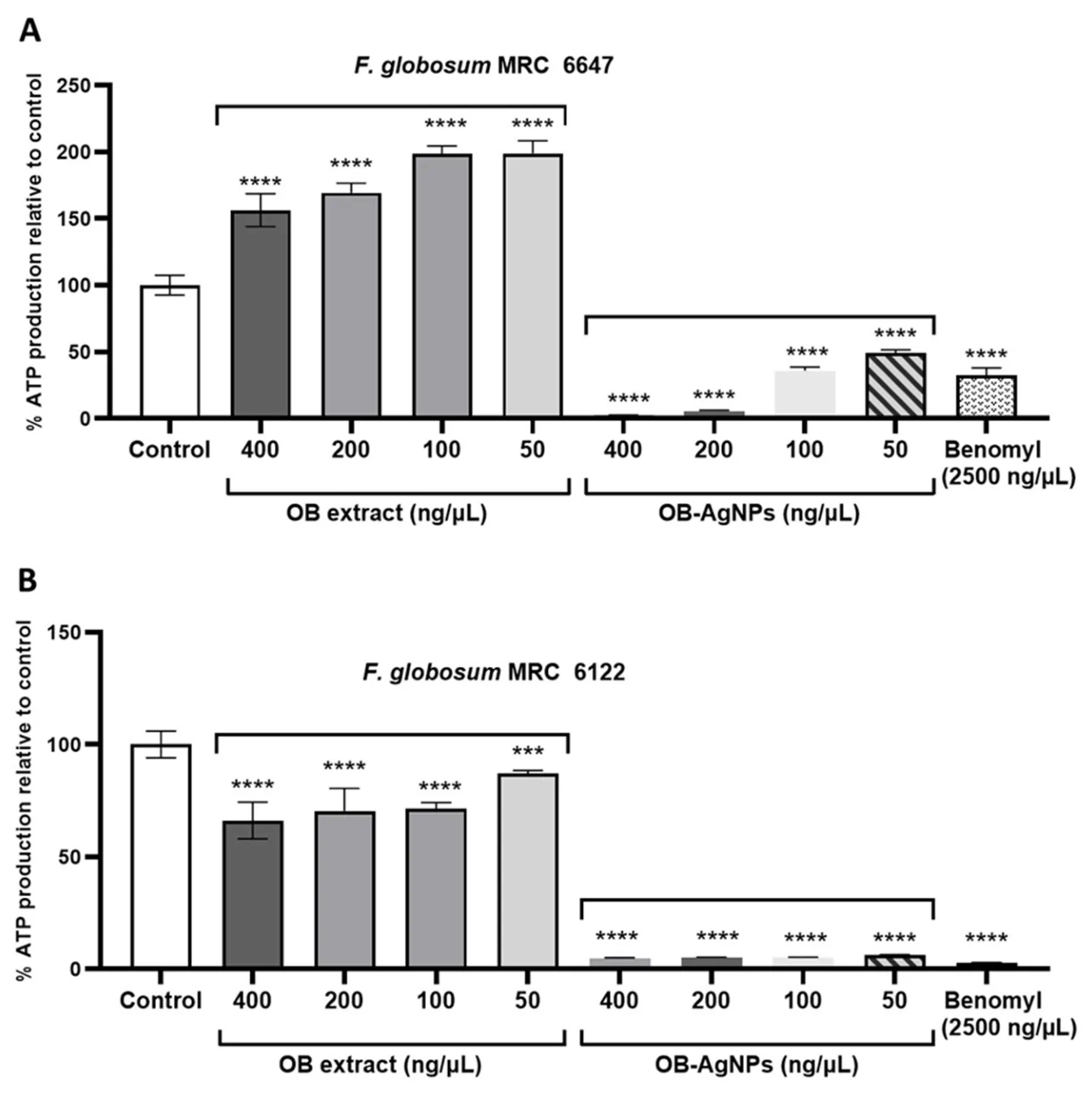
Conidial viability [percent adenosine triphosphate (ATP) production relative to unexposed control] of *Fusarium globosum* (**A**) MRC 6647 and (**B**) MRC 6122 after 96 h exposure to *Ocimum basilicum* extract (OB extract) and the biosynthesized silver nanoparticles (OB-AgNPs). Data are presented relative to the unexposed control. Statistical differences are indicated as *** *p* < 0.001 and **** *p* < 0.0001.

The effects of *O. basilicum*, along with its OB-AgNPs on *Fusarium* conidial germination and growth were assessed by measuring the percentage of adenosine triphosphate (adenosine 5′-triphosphate, ATP) production relative to unexposed controls. ATP levels serve as a proxy for metabolic activity and, by extension, spore viability. Benomyl, a broad-spectrum fungicide, was included as a positive control. The *Fusarium* MRC strains tested included *F*. *verticillioides*, *F. proliferatum*, *F. subglutinans*, *F. graminearum*, and *F. globosum* following 96 h of exposure. The growth inhibitory effects of the extract and OB-AgNPs against their respective *Fusarium* strains at varying concentrations are comprehensively presented in Table S2, as well as in Figure 6, Figure 7, Figure 8, Figure 9 and Figure 10.

Across most strains, except for *F. globosum* MRC 6122 (Figure 10B), treatment with the crude extract showed negligible inhibitory effects and, in most cases, even stimulated ATP production indicating possible stimulation of metabolic activity. The *O. basilicum* extract significantly stimulated ATP production in all strains treated with 50 ng/µL (Figure 6, Figure 7, Figure 8, Figure 9 and Figure 10) except for *F. globosum* MRC 6122 (Figure 10B), where a significant inhibitory effect (87% ATP production compared to the control) was observed. At a treatment concentration of 100 ng/µL, no effect was observed in the ATP percentage in *F. verticillioides* MRC 826-E (Figure 6A) and *F. proliferatum* MRC 8550 (Figure 7A). A significant inhibitory effect was observed in *F. globosum* MRC 6122 (71% ATP production) (Figure 10B), and a stimulating effect was observed in all the other *Fusarium* strains (Figure 6, Figure 7, Figure 8, Figure 9 and Figure 10). A similar trend was observed at the 200 and 400 ng/µL exposure of the fungal spores to the *O. basilicum* extract where the extract had no effect on *F. verticillioides* MRC 826-E (Figure 6A) and MRC 826-J (Figure 6B) as well as on both strains of *F. proliferatum* (Figure 7) and 400 ng/µL also had no effect on the % ATP production in *F. subglutinans* MRC 8553 but 200 ng/µL significantly stimulated ATP production in this strain. A stimulatory effect in ATP production was observed in the *F*. *verticillioides* MRC 8559 (Figure 6C), *F. subglutinans* MRC 8554 (Figure 8B), *F. graminearum* MRC 6010 (Figure 9) as well as *F. globosum* MRC 6647 (Figure 10A) exposed to 400 and 200 ng/µL, and a significant inhibitory effect was observed in *F. globosum* MRC 6122 (Figure 10B) exposed to both concentrations of the *O. basilicum* extract.

The OB-AgNPs, particularly at concentrations of 400, 200, and 100 ng/µL, exhibited a significant inhibitory effect on conidial viability, as measured by ATP production, in all tested *Fusarium* strains (Table S2; Figure 6, Figure 7, Figure 8, Figure 9 and Figure 10), except for the two *F. proliferatum* strains (Figure 7). In *F. proliferatum* MRC 8550, OB-AgNPs led to a concentration-dependent reduction in ATP production, resulting in viability levels of 70.1%, 72.9%, 80.6%, and 91.8% at 400, 200, 100, and 50 ng/µL, respectively (Figure 7A). In contrast, for *F. proliferatum* MRC 8549, a significant reduction in ATP production was observed only at the highest concentration of 400 ng/µL (82.3%), with no appreciable effect at lower concentrations (Figure 7B).

### 2.5. Morphological Effect of Ocimum basilicum Extract and the Biosynthesized Silver Nanoparticles on Fusarium Conidia

**Figure 11:**
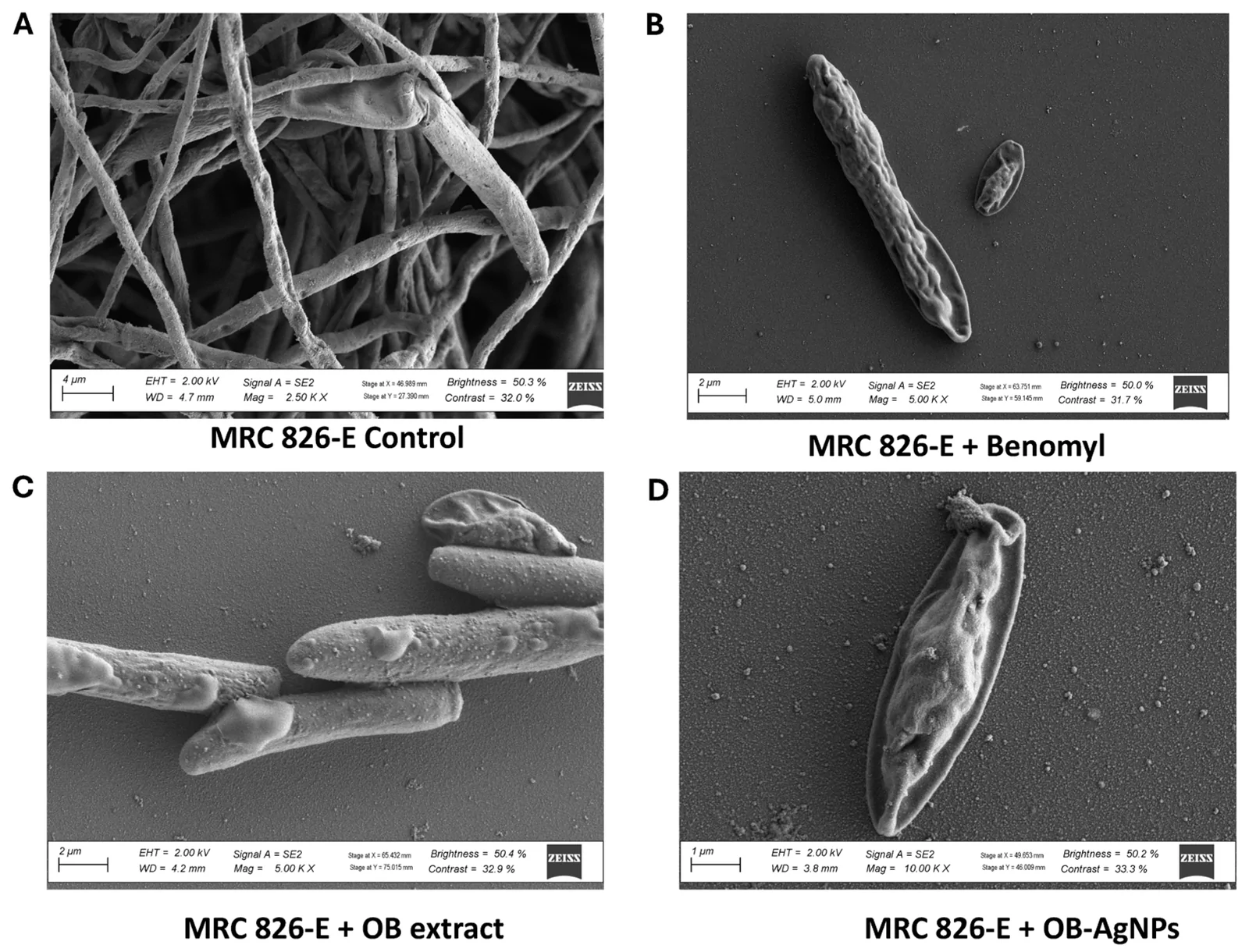
Scanning electron microscopy (SEM) images of *Fusarium verticillioides* MRC 826-E conidia and hyphae following 96 h exposure to 50 ng/µL *Ocimum basilicum* extract (OB extract) and the biosynthesized silver nanoparticles (OB-AgNPs). Fungal cultures were incubated in potato dextrose medium with or without treatment: (**A**) untreated control; (**B**) Benomyl (2.5 mg/mL); (**C**) *Ocimum basilicum* extract; and (**D**) OB-AgNPs treatment.

**Figure 12:**
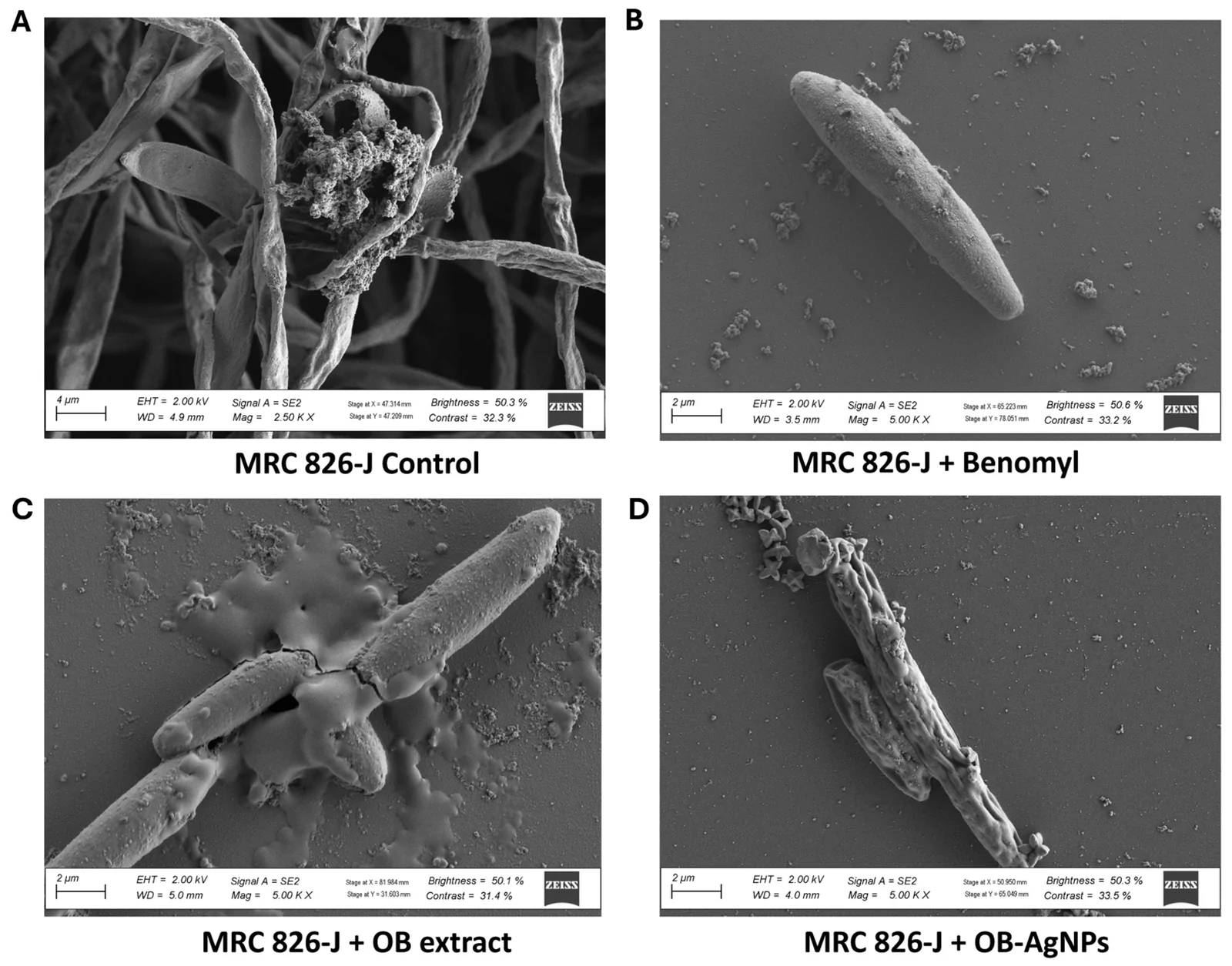
Scanning electron microscopy (SEM) images of *Fusarium verticillioides* MRC 826-J conidia and hyphae following 96 h exposure to 50 ng/µL *Ocimum basilicum* extract (OB extract) and the biosynthesized silver nanoparticles (OB-AgNPs). Fungal cultures were incubated in potato dextrose medium with or without treatment: (**A**) untreated control; (**B**) Benomyl (2.5 mg/mL); (**C**) *Ocimum basilicum* extract (OB extract); and (**D**) the biosynthesized silver nanoparticles (OB-AgNPs) treatment.

**Figure 13:**
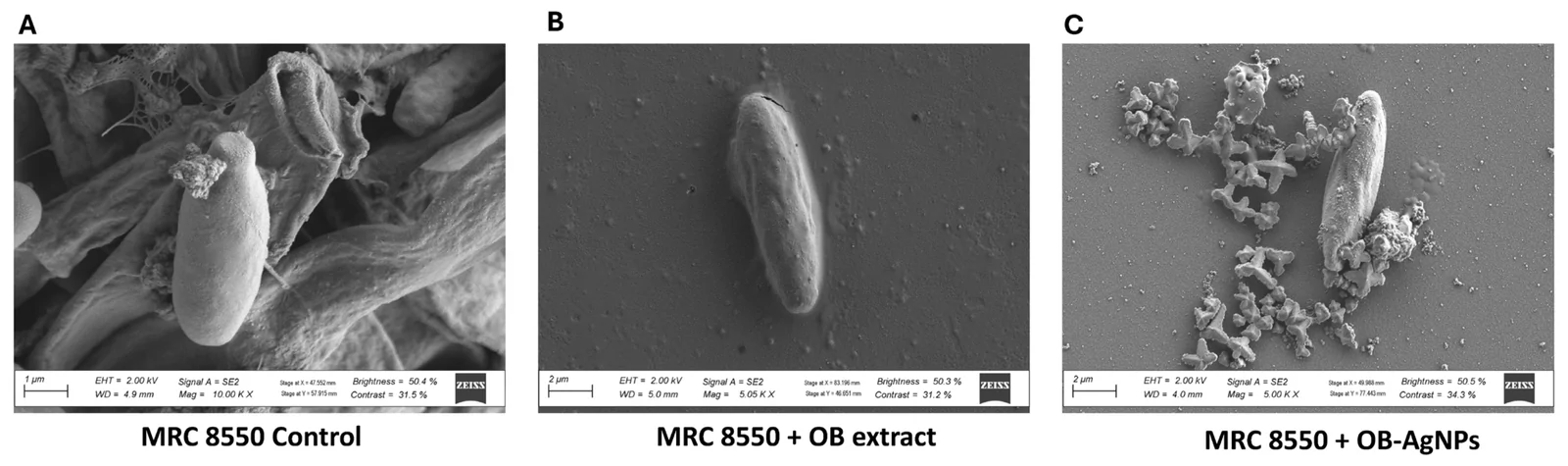
Scanning electron microscopy (SEM) images of *Fusarium proliferatum* MRC 8550 conidia and hyphae following 96 h exposure to 50 ng/µL *Ocimum basilicum* (OB) extract and silver nanoparticles (OB-AgNPs). Fungal cultures were incubated in potato dextrose medium with or without treatment: (**A**) untreated control; (**B**) *Ocimum basilicum* extract (OB extract); and (**C**) the biosynthesized silver nanoparticles (OB-AgNPs) treatment.

**Figure 14:**
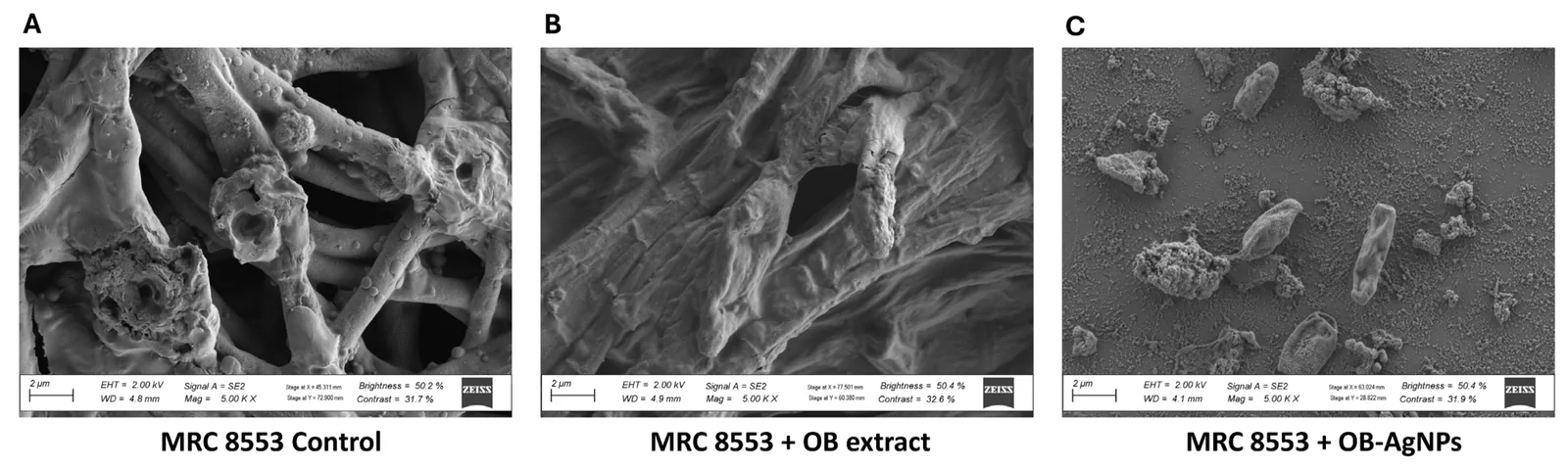
Scanning electron microscopy (SEM) images of *Fusarium subglutinans* MRC 8553 conidia and hyphae following 96 h exposure to 50 ng/µL *Ocimum basilicum* (OB) extract and silver nanoparticles (OB-AgNPs). Fungal cultures were incubated in potato dextrose medium with or without treatment: (**A**) untreated control; (**B**) *Ocimum basilicum* extract (OB extract); and (**C**) the biosynthesized silver nanoparticles (OB-AgNPs) treatment.

**Figure 15:**
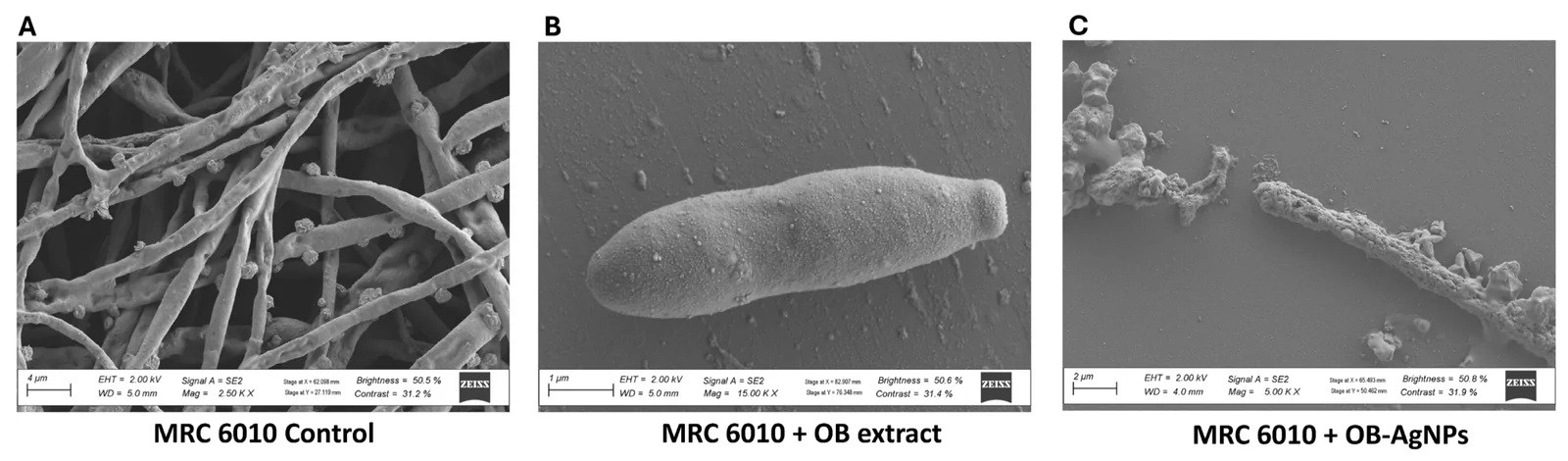
Scanning electron microscopy (SEM) images of *Fusarium graminearum* MRC 6010 conidia and hyphae following 96 h exposure to 50 ng/µL *Ocimum basilicum* (OB) extract and silver nanoparticles (OB-AgNPs). Fungal cultures were incubated in potato dextrose medium with or without treatment: (**A**) untreated control; (**B**) *Ocimum basilicum* extract (OB extract); and (**C**) the biosynthesized silver nanoparticles (OB-AgNPs) treatment.

**Figure 16:**
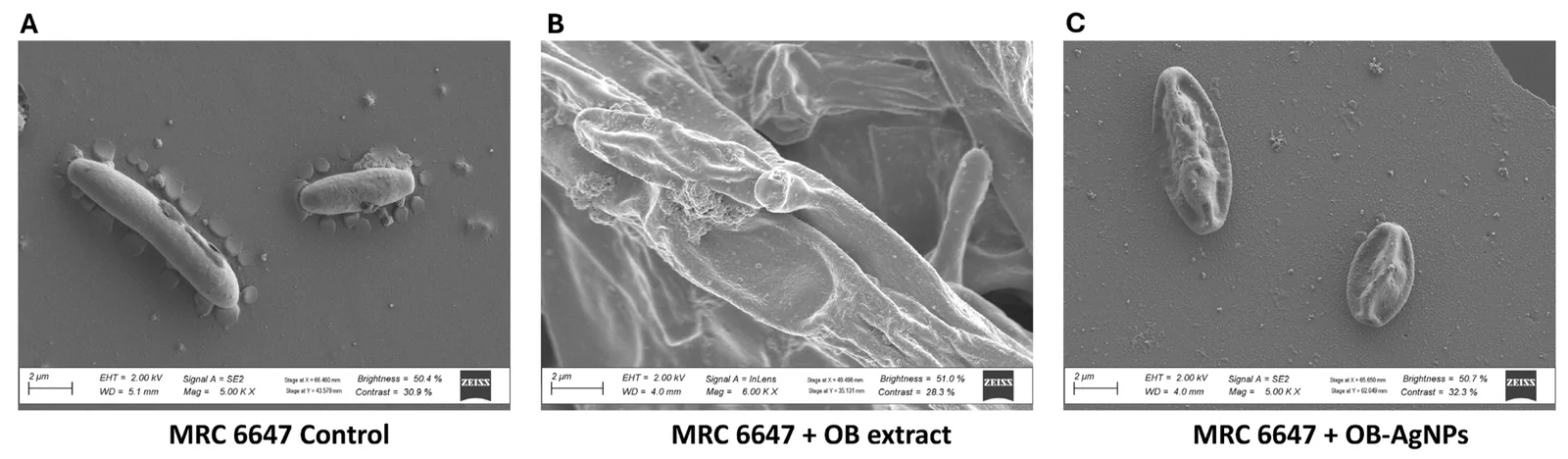
Scanning electron microscopy (SEM) images of *Fusarium globosum* MRC 6647 conidia and hyphae following 96 h exposure to 50 ng/µL *Ocimum basilicum* (OB) extract and silver nanoparticles (OB-AgNPs). Fungal cultures were incubated in potato dextrose medium with or without treatment: (**A**) untreated control; (**B**) *Ocimum basilicum* extract (OB extract); and (**C**) the biosynthesized silver nanoparticles (OB-AgNPs) treatment.

Based on the antifungal susceptibility assays, the *O. basilicum* crude extract and its corresponding OB-AgNPs were selected at a concentration of 50 ng/µL for further imaging analysis, as this represented the lowest concentration at which strong antifungal activity was observed for the OB-AgNPs. Both treated conidia (exposed to the extract or AgNPs) and untreated controls were examined to assess structural alterations induced by the treatments. The selected untreated conidia of MRC strains, including *F. verticillioides* MRC 826-E and MRC 826-J, *F. proliferatum* MRC 8550, *F. subglutinans* MRC 8553, *F. graminearum* MRC 6010, and *F. globosum* MRC 6647, exhibited typical spore growth after 96 h of incubation (Figure 11, Figure 12, Figure 13, Figure 14, Figure 15 and Figure 16). These conidia developed into long, interconnected hyphal chains that appeared morphologically regular and structurally intact. Scanning electron microscopy (SEM) analysis revealed distinct morphological differences in the control *F. verticillioides* MRC 826-E conidia and hyphae (Figure 11A) following treatment with the positive control, Benomyl (Figure 11B), the *O. basilicum* extract (Figure 11C) and the OB-AgNPs (Figure 11D). The Benomyl-treated conidia displayed pronounced morphological alterations, including shrinkage, surface wrinkling, and collapse of cellular structure, indicative of strong antifungal activity (Figure 11B). Conidia exposed to the crude extract showed little deformation (Figure 11C). Notably, OB-AgNPs treatment caused the most severe morphological damage; conidia appeared extensively collapsed, with highly disrupted and roughened surfaces, suggesting significant loss of cellular integrity and leakage of intracellular contents (Figure 11D). These observations confirm the potent antifungal effect of OB-AgNPs, surpassing both the crude extract and Benomyl in terms of structural impact on fungal conidia. SEM analysis of *F. verticillioides* MRC 826-J conidia revealed marked morphological differences between treated and untreated samples. In the untreated control (Figure 12A), the Benomyl-treated conidia (Figure 12B) and the conidia treated with 50 ng/µL of the *O. basilicum* extract (Figure 12C), the fungal structures appeared healthy, with smooth, intact hyphae and conidia exhibiting normal architecture. The most severe structural damage was observed in the OB-AgNPs-treated conidia (Figure 12D), which showed extensive shrinkage, deformation, and surface disintegration. For *F. proliferatum* MRC 8550 (Figure 13), *F. subglutinans* MRC 8553 (Figure 14), *F. graminearum* MRC 6010 (Figure 15), and *F. globosum* MRC 6647 (Figure 16), a consistent trend was observed in the SEM analyses. Treatment with crude extract at 50 ng/µL (Figure 13B, Figure 14B, Figure 15B and Figure 16B) did not result in any visible alterations to conidial structure when compared to the untreated controls (Figure 13A, Figure 14A, Figure 15A and Figure 16A), and the conidia retained their normal morphology. This lack of morphological disruption corresponded with the high percentage of ATP production recorded for these strains following *O. basilicum* extract exposure, indicating limited antifungal effect. In the case of OB-AgNPs treatment, *F. proliferatum* MRC 8550 (Figure 13C) similarly exhibited no significant structural damage to the conidia, which is consistent with the minimal reduction in ATP production observed for this strain. However, for *F. subglutinans* MRC 8553 (Figure 14C), *F. graminearum* MRC 6010 (Figure 15C), and *F. globosum* MRC 6647 (Figure 16C), exposure to OB-AgNPs resulted in severe morphological damage. SEM images revealed notable conidial shrinkage and surface collapse, reflecting the strong antifungal activity of the nanoparticles in these strains, as also indicated by the marked reduction in ATP production.

## 3. Discussion

Despite their attractive properties, metallic nanoparticles have limited biological applications due to toxicity concerns, often intensified by hazardous chemical stabilizers. Green nanotechnology, particularly plant-mediated synthesis, plays a key role in eliminating environmental hazards associated with the chemical industry by replacing toxic reagents with eco-friendly alternatives. Plant phytochemicals act as natural reducing and capping agents, enabling a safe, sustainable, and efficient synthesis of AgNPs. *Ocimum basilicum* is a well-known medicinal plant with a wide spectrum of biological activities [47,48].

Chemical synthesis of metal nanoparticles typically relies on external reducing and capping agents to control growth, prevent aggregation, and ensure stability. In this study, *O. basilicum* extract was successfully utilized as a dual reducing and stabilizing agent, leveraging its rich secondary metabolites to biosynthesize AgNPs. Building on our previous high-throughput screening of South African plants using a rapid microtiter plate method [49], this work optimized extract concentrations to yield the smallest, most stable particles. Furthermore, because elevated temperatures favor smaller particle diameters [50], synthesis was conducted at 70 °C. The resulting OB-AgNPs were thoroughly characterized for morphology, size distribution, and surface charge, and their antifungal efficacy was evaluated against various *Fusarium* species.

The chemistry of the plant is well-documented and reported to have interesting metabolites such as, *inter alia*, rosmarinic, caffeic, and chicoric acids in addition to different anthocyanins. The LC-MS analysis of the aqueous extract exhibited 10 major peaks in ES negative mode (Figure 1 and Figure S2 and Table S1). The compounds were tentatively identified as syringic acid, caftaric acid, aralidioside, caffeic acid, vicenin-2, chicoric acid, rutin, sagerinic acid, nicotiflorin, and rosmarinic acid. Rosmarinic acid, vicenin-2, and chicoric acid are the major constituents present in the extract (Table S1) [46,47,48,49,50,51,52]. These compounds are common phenolic constituents in many members of the *Ocimum* genus and generally water-soluble.

The LC-MS literature profiling confirms that *O. basilicum* is characterized by caffeic acid derivatives, with rosmarinic and chicoric acids anchoring the phenolic profile [53,54,55]. Other reports note significant concentrations of caffeic acid, quercetin, caffeic acid ethyl ester, and neopetoidin B [56]. While total extract preparations can be highly concentrated in vicenin-2, the matrix exhibits a diverse flavonoid assembly featuring vitexin and apigenin derivatives [55,57]. This structural variation aligns perfectly with our experimental results presented in Table S1 and Figure 1.

The visual observation of the color changes from light yellow to brown color for the silver salt/plant extract mixtures after the 1 h incubation (Figure S1) gave an indication about the formation of the AgNPs. This confirmed the ability of phytochemicals in *O. basilicum* aqueous extract to reduce the Ag^+^ ions to Ag^0^. The cause of this brown color in the AgNPs’ colloidal solution is a result of the oscillation of free conduction electrons known as SPR. This phenomenon can be manifested with a maxima absorbance between 400 and 500 nm in the UV-Vis spectra of the AgNPs. Figure 2 shows the UV-Vis spectra of OB-AgNPs exhibited a maximum absorbance of ~437 nm.

TEM analysis revealed well-defined, quasi-spherical AgNPs with no significant agglomeration (Figure S3). The nanoparticles exhibited sizes ranging from 18.5 to 37.4 nm. The observed size variation is attributed to the presence of diverse plant metabolites with different reductions and capping kinetics, as commonly reported for plant-mediated synthesis. DLS analysis provided complementary information on nanoparticle stability, size distribution, and hydrodynamic diameter (Table 1, Figure 3). The OB-AgNPs showed a zeta potential of ~−16.3 mV, which falls within the reported range for stable, non-agglomerated biosynthesized nanoparticles. The polydispersity index (PDI) value of 0.361 indicated a moderate disperse particle distribution. The hydrodynamic diameter (57.05 nm) closely matched the mean particle size observed by TEM, confirming consistency between the two techniques.

The crystalline nature of the OB-AgNPs was confirmed by X-ray diffraction analysis. Four characteristic Bragg peaks were observed at 2θ values of 38.185°, 44.393°, 64.578°, and 77.549°, corresponding to the (111), (200), (220), and (311) planes of face-centered cubic metallic silver (Figure S4). These reflections are consistent with the Joint Committee on Powder Diffraction Standards (JCPDS, silver card no. 04-0783). Minor additional peaks were attributed to traces of silver chloride and/or residual plant-derived components remaining after synthesis. The formation of AgCl is plausible because chloride ions naturally present in the plant extract may react with a small fraction of Ag^+^ ions during the reduction process.

The dimensional metrics of the OB-AgNPs varied by technique (TEM: 18.5–37.4 nm; DLS: 57.08 nm; XRD: 11.18 nm), a trend widely observed in biogenic synthesis due to differing measurement principles [7,58]. The larger DLS hydrodynamic size stems from the hydrated organic corona of *O. basilicum* phytochemicals surrounding the core, mirroring major discrepancies seen in *Ferula gummosa* (TEM: 5.63 nm vs. DLS: 111.5 nm) [59], *Agave potatorum* [58], and *Cyperus rotundus* [60]. Conversely, TEM captures the physical core in a dehydrated state [59]. The smaller XRD value (11.18 nm) reflects the individual monocrystalline scattering domains calculated via the Debye–Scherrer equation rather than the physical particle boundary [7]. This aligns with *Diospyros paniculata* syntheses where XRD tracked core crystallite structures independently of capping effects [7].

**Figure 17:**
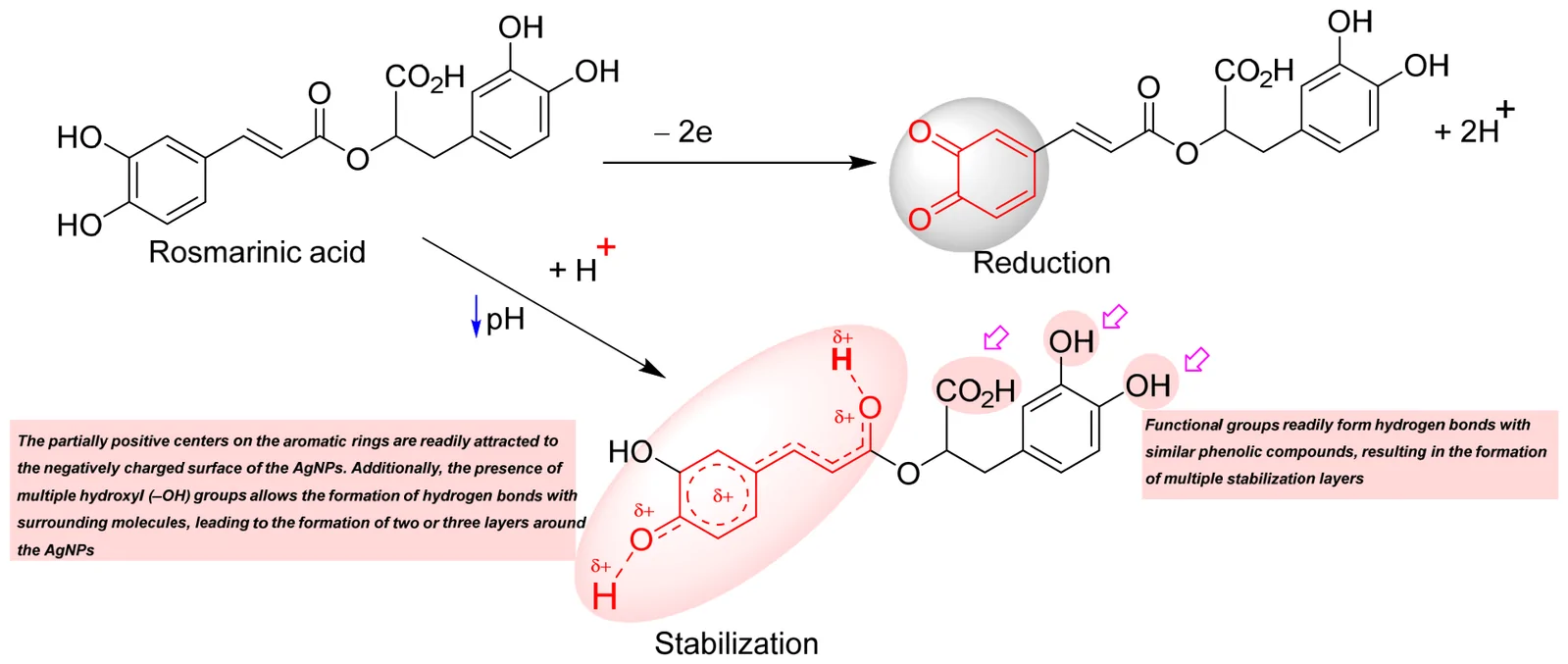
Chemical structures illustrating the possible reduction and stabilization mechanisms of phenolic constituents in *Ocimum basilicum* aqueous extract (rosmarinic acid used as a representative example).

To identify the functional groups involved in the reduction and stabilization of the nanoparticles, FTIR spectroscopy was used to compare the intact plant extract with the synthesized OB-AgNPs (Figure 4). The presence of major absorption bands in the extract at 1050–1076 cm^−1^ (C–O, phenolic), 1616 cm^−1^ (C=O of α,β-unsaturated ketones/carboxylic groups, and C=C aromatic), 2950–3000 cm^−1^ (C–H, aromatic), and 3300–3340 cm^−1^ (O–H stretching) confirmed the primary role of phenolic and related phytochemicals in the synthesis. Following nanoparticle formation, pronounced shifts and intensity changes were observed in these bands—most notably the decreased intensity of the peaks at 2973, 1379, and 1066 cm^−1^, alongside the appearance of a distinct peak at 1616 cm^−1^. These spectroscopic modifications indicate the robust conjugation of the aromatic systems onto the nanoparticle surface, aligning perfectly with the proposed molecule–surface interaction mechanism (Figure 17) [7]. Specifically, direct coordination is evidenced by downshifts in the carbonyl (C=O) stretching bands due to silver surface binding [58], while the decreased intensity of phenolic hydroxyl signals, accompanied by the emergence of new metal–oxygen vibrational peaks at 400–600 cm^−1^, reflects the localized consumption of the plant’s reducing agents and subsequent Ag–O bond stabilization [61].

The in vitro stability of OB-AgNPs was evaluated in potato dextrose broth containing 1% DMSO and incubated for up to 24 h, with absorbance monitored by UV–Vis spectroscopy at 0, 12, and 24 h. The selected time interval reflects typical preparation and application durations. Stable nanoparticles are expected to retain absorption maxima similar to the control samples. As shown in Figure 5A,B, the AgNPs maintained a stable surface plasmon resonance band at 437 nm over 24 h, indicating good stability under the tested conditions. Additionally, stability evaluations were performed on a sample stored at room temperature for two years. The recent UV-Vis spectra, ZP, and hydrodynamic size distribution measurements remained remarkably similar to the initial data (Figure 5C and Figure S5). This long-term stability indicates the exceptional capability of the capping phytochemicals to stabilize the OB-AgNPs and prevent agglomeration over extended periods.

The identification of prominent phenolic compounds, specifically vicenin-2, chicoric acid, and rosmarinic acid (Figure 1) [46,51,52], underpins the formation of stable, bioactive nanoparticle conjugates. Owing to their phenolic architecture and abundant hydrogen donor/acceptor sites, these pure small molecules are ideally suited to drive the reduction of AgNO_3_ and cap the resulting AgNPs. A plausible reduction and stabilization mechanism for these constituents is illustrated in Figure 17. The proposed pathway posits that phenolic hydroxyl groups mediate the reduction of Ag^+^ to Ag^0^. Concurrently, the peripheral carboxylic groups, alongside positive localized charges induced by phenolic hydroxyl protonation, establish dynamic centers that electrostatically protect and stabilize the synthesized nanoparticles.

Recently plant-mediated AgNPs have been reported with notable antifungal activity against various *Fusarium* species, although their effectiveness varies depending on the plant source and concentration used. AgNPs synthesized using *Pongamia pinnata* exhibited strong efficacy against *F. oxysporum*, achieving up to 95.4% inhibition at 150 µg/mL under in vitro conditions, while 100 µg/mL was identified as the optimal concentration under greenhouse and field conditions [62]. The present study evaluated the antifungal efficacy of *O. basilicum* aqueous extract and OB-derived AgNPs against selected *Fusarium* species associated with mycotoxin contamination and crop losses. The findings demonstrate that while the crude extract exhibited limited inhibitory activity, the biosynthesized OB-AgNPs showed pronounced and broad-spectrum antifungal effects against most tested strains. The concentration range evaluated for the aqueous *O. basilicum* extract (50–400 ng/µL) was deliberately selected to enable a direct comparison with the corresponding biosynthesized OB-AgNPs rather than to establish the minimum inhibitory concentration of the crude extract. Consequently, the extract results should be interpreted within the context of this comparative study design. The ATP-based viability assays revealed that exposure to crude extract generally resulted in minimal inhibition and, in several cases, stimulated fungal metabolic activity. This stimulatory effect may be attributed to the presence of phytochemicals and organic compounds in the crude extract that could serve as alternative carbon sources or metabolic substrates for *Fusarium* species. Similar growth-promoting effects of plant extract at sub-inhibitory concentrations have been reported in previous studies, suggesting that low concentrations of bioactive compounds may enhance fungal metabolism rather than suppress it [63]. However, because only a limited concentration range was investigated, it is not possible to conclude that this response represents hormesis or a biphasic dose–response relationship. Confirmation of such a response would require evaluation across a substantially broader concentration range. These observations indicate that, within the concentration range evaluated, the crude extract exhibited limited antifungal activity compared with the corresponding biosynthesized OB-AgNPs [35].

The OB-AgNPs exhibited strong inhibitory activity against most *Fusarium* strains at concentrations of 50–400 ng/µL, as reflected by significant reductions in ATP production. The enhanced antifungal performance of the nanoparticles can be attributed to their small size, increased surface area, and improved bioavailability, which facilitates close interaction with fungal cell membranes [64,65,66]. Silver nanoparticles are known to induce oxidative stress, disrupt membrane integrity, interfere with enzyme activity, and impair DNA replication, ultimately leading to cell death [67]. The green synthesis approach employed in this study may have further enhanced nanoparticle stability and bioactivity through capping by phytochemicals present in the *O. basilicum* extract [68].

Notably, strain-dependent variability in susceptibility to OB-AgNPs was observed. While most strains showed marked sensitivity, *F*. *proliferatum* MRC 8550 and MRC 8549 exhibited comparatively lower inhibition, particularly at lower concentrations. This reduced susceptibility may reflect inherent physiological differences, variations in cell wall composition, or differential activation of stress–response pathways. Such strain-specific responses have been documented in previous antifungal studies [69] and highlight the importance of evaluating multiple isolates when assessing antimicrobial efficacy. AgNPs synthesized from *Pedalium murex* leaf extract demonstrated antifungal activity against *F. graminearum*, producing an inhibition zone of approximately 11 mm at 80 µg/mL [70]. In contrast, *Blumea sinuata*-derived AgNPs required higher concentrations, with 1.5 mg/mL achieving around 50.45% growth inhibition of *F. oxysporum* [71]. Likewise, AgNPs synthesized from *Solanum nigrum* leaf extract showed moderate antifungal activity against *F. circinatum*, with approximately 56.66% inhibition observed at 75 µg/mL [72]. Other plant systems demonstrated activity at comparatively higher doses. For example, *Anchusa arvensis*-mediated AgNPs exhibited antifungal effects against *F. solani*, producing an inhibition zone of about 11.2 mm at 20 mg/mL [73]. Similarly, *Chenopodium album*-derived AgNPs were effective against *F. oxysporum* at 800 µg/mL [74]. In addition, AgNPs synthesized using *O. basilicum* extract in a nanozyme system showed strong antifungal activity against *F. pseudograminearum*, effectively inhibiting both mycelial growth and spore development, although the exact effective concentration was not explicitly reported [75].

Scanning electron microscopy further corroborated the biochemical findings by revealing extensive structural damage in OB-AgNP-treated conidia. Severe morphological alterations, including surface collapse, shrinkage, and membrane disruption, were evident in most strains following nanoparticle exposure. These changes are indicative of compromised cell wall integrity and leakage of intracellular contents, supporting the hypothesis that OB-AgNPs exert their antifungal effects primarily through physical and biochemical damage to cellular structures. In contrast, conidia treated with *O. basilicum* crude extract largely retained normal morphology, consistent with their limited inhibitory effects observed in the ATP assays. The selection of 50 ng/µL for imaging analysis proved valuable in demonstrating that even the lowest effective nanoparticle concentration was capable of inducing pronounced ultrastructural damage in sensitive strains. This finding highlights the high potency of OB-AgNPs and suggests that effective antifungal activity may be achieved at relatively low dosages, thereby reducing potential environmental and toxicological risks associated with higher nanoparticle concentrations.

Comparatively, the antifungal activity of OB-AgNPs in this study aligns with previous reports on green-synthesized AgNPs derived from medicinal plants, which have demonstrated strong inhibitory effects against *Fusarium* and other phytopathogens [76]. The superior performance of OB-AgNPs relative to both crude extract and the commercial fungicide Benomyl in certain strains further underscores their potential as alternative or complementary disease-control agents. Given the growing concerns surrounding chemical fungicide resistance, environmental contamination, and human health risks, plant-mediated nanoparticle synthesis represents a promising sustainable approach to crop protection. Nevertheless, some limitations should be acknowledged.

The study was conducted under in vitro conditions, which may not fully replicate the complexity of field environments. Factors such as soil composition, microbial interactions, and environmental stressors may influence nanoparticle stability and efficacy in situ. Additionally, long-term ecotoxicological impacts and potential accumulation of silver nanoparticles in agricultural ecosystems require careful evaluation before large-scale application.

## 4. Materials and Methods

### 4.1. Chemicals and Reagents

Various chemicals used in this study were purchased from Sigma-Aldrich (Merck KGaA, Darmstadt, Germany), including Benomyl (CAS No. 17804-35-2), bisbenzimide Hoechst 33342 trihydrochloride (CAS No. 875756-97-1). Dimethyl sulfoxide (DMSO), sucrose, magnesium sulphate (MgSO_4_. 7H_2_O), zinc sulphate (ZnSO_4_), potassium chloride (KCl), potassium dihydrogen phosphate (KH_2_PO_4_), calcium nitrate [Ca(NO_3_)_2_], ferric chloride (FeCl_3_), potato dextrose broth (PDB), silver nitrate (AgNO_3_), and glycine (Gly) purchased from Merck (KGaA, Darmstadt, Germany). The BacTiter-Glo™ (ADG8232) Luminescent Microbial Cell Viability Assay kit was obtained from Anatech (Promega, Madison, WI, USA). N-Acetyl-L-cystine was purchased from Boehringer Mannheim GmbH (Mannheim, Germany). Polystyrene 96-well microtiter plates were obtained from Greiner Bio-One GmbH (Frickenhausen, Germany).

### 4.2. Equipment

The SPECTROstar Nano (BMG LABTECH, Ortenberg, Germany) 2450 ultraviolet/visible (UV-Vis) spectrophotometer was used to monitor the characteristic peaks of the AgNPs. High-resolution transmission electron microscopy (HRTEM) (FEI Tecnai G2 F20 S-Twin, FEI Company, Hillsboro, OR, USA, operated at 200 kV) was employed to study the morphology of the AgNPs. Field Emission Scanning Electron Microscope (Miramar, Oxfordshire, UK). Dynamic light scattering (DLS) analysis was conducted using a Malvern Zetasizer Instrument (Malvern Panalytical Ltd., Malvern, UK). The IR spectra were measured using a Fourier-transform infrared (FTIR) spectrophotometer (PerkinElmer Spectrum 100, Llantrisant, Wales, UK). Lyophilization was carried out using a bench-top freeze dryer equipped with an external rotary vacuum pump (ANCHOR, Shanghai Scientific Instrument, Shanghai, China). X-ray diffraction (XRD) patterns were collected using a Bruker AXS D8 advance diffractometer with Cu-Kα_1_ radiation (λ = 1.5406 Å) to determine the crystallinity of the AgNPs. The separation of the nanoparticles was conducted using Sigma 2-16P (Sigma Laborzentrifugen GmbH, Osterode am Harz, Germany) centrifuge at 12,000 rpm.

### 4.3. Preparation of the Plant Extract

The aerial parts of *O. basilicum* were collected from the Cape Peninsula University of Technology (CPUT), Bellville Campus nursery ($-33.9322^{\circ }/18.6419^{\circ }$), in October 2022. A voucher herbarium specimen (NAT-02-22) was authenticated by the Department of Horticultural Sciences at CPUT. The plant material was dried under shade at room temperature, pulverized to a fine powder using a mechanical grinder, and passed through a 1.0 mm sieve to ensure uniform particle distribution. The aqueous extract of *O. basilicum* was prepared by mixing 50 g of the powdered plant material with 500 mL of boiled distilled water and shaking for one hour. The mixture was allowed to cool to room temperature, decanted and centrifuged at 4000 rpm for 30 min. The resulting supernatant was then filtered using 0.25 µm syringe filter. The filtrate was frozen at −80 °C for 48 h and subsequently freeze-dried. Lyophilization was performed at a condenser temperature of −85 °C and a chamber pressure of 7–10 Pa for 48 h. The dried extract (4.7 g) was stored in a desiccator at room temperature for further experiments.

### 4.4. Liquid Chromatography–Mass Spectrometry (LC-MS) Analysis

A Waters Cyclic Quadrupole time-of-flight (qTOF) mass spectrometer (MS) connected to a Waters Acquity i-Class ultra-performance liquid chromatograph (UPLC) (Waters, Milford, MA, USA) was used for high-resolution UPLC-MS analysis. Electrospray ionization in negative mode was used, with a cone voltage of 20 V, desolvation temperature of 275 °C, desolvation gas at 600 L/h, and the rest of the MS settings optimized for the best resolution and sensitivity. Data were acquired by scanning from m/z 100 to 1200 m/z in resolution mode as well as in MSE mode. In MSE mode, two channels of MS data were acquired, one at a low collision energy (6 V) and the second using a collision energy ramp (25−100 V) to obtain fragmentation data as well. Leucine enkephalin was used as lock mass (reference mass) for accurate mass determination and the instrument was calibrated with sodium formate. Separation was achieved on a Waters HSS T3, 2.1 × 150 mm, 1.8 μm column. An injection volume of 0.5 μL was used and the mobile phase consisted of 0.1% formic acid (solvent A) and acetonitrile containing 0.1% formic acid as solvent B. The gradient started at 100% solvent A for 1 min and changed to 28% B over 7 min in a linear way, followed by 44%B after 10 min and 100% B after 12 min, with a wash step of 1 min at 100% B. After returning to 0% B, the column was re-equilibrated at initial conditions for 2 min. The flow rate was 0.35 mL/min, and the column temperature was maintained at 60 °C. Quantification of the metabolites was carried out in a semi-quantitative manner using a calibration curve constructed from rosmarinic acid calibration standards ranging from 1 to 10 mg/L, allowing for the relative estimation of compound concentrations.

### 4.5. Synthesis of the Silver Nanoparticles (OB-AgNPs)

The synthesis of AgNPs was optimized using a high-throughput, micro-volume screening protocol in 96-well plates according to our previously reported method [49]. A fresh 32.0 mg/mL stock solution of the aqueous extract was prepared in distilled water, followed by serial dilutions ranging from 0.5 to 16.0 mg/mL in a 96-well plate. To each well containing 50 μL of extract solution, 250 μL of 0.3 mM silver nitrate solution was added. The reaction mixtures were incubated at 70 °C. Nanoparticle synthesis was initially monitored by a color change from light yellow to brown and confirmed via UV-Vis spectrophotometry [49].

The optimal concentration of the plant extract required to successfully reduce the silver salt was determined to be 2.0 mg/mL, selected based on the peak shape and SPR band intensity. Following optimization, this concentration was scaled up to yield enough AgNPs for subsequent physical characterization and in vitro bioassays. The optimized parameters for the scaled-up synthesis were established as follows: 15 mL of a 0.30 mM AgNO_3_ solution was mixed with 10.0 mL of the aqueous plant extract (at the optimized concentration of 2.0 mg/mL) and incubated at 70 °C for 1 h. The same ratio was then used on a larger scale to prepare a greater quantity. Purification of the nanoparticles was achieved by centrifugation at 12,000 rpm. The residue was washed with distilled water and subjected to a second cycle of centrifugation (12,000 rpm) to eliminate completely the unreacted precursors and unbound phytochemicals, followed by freeze-drying of the final pellets.

### 4.6. Characterizations of the Biosynthesized Silver Nanoparticles (OB-AgNPs)

The synthesized nanoparticles were characterized using a suite of analytical techniques.

The surface plasmon resonance absorption peak characteristic of metal nanoparticles was monitored using a microtiter plate UV/Vis reader. The DLS technique provides the zeta potential which indicates the potential stability of the particles in a colloidal system. This was done using a Malvern Zetasizer Instrument with Zetasizer software version 7.11 at 25° and a 90° angle.

For high-resolution transmission electron microscopy (HRTEM) analysis, samples were prepared by drop-coating a single drop of the respective nanoparticle solution onto a holey carbon-coated copper grid. The grids were then dried under a Xenon lamp for 10 min prior to microscopic examination. Micrographs were acquired in bright-field mode at an accelerating voltage of 200 kV.

The crystallinity of the samples was then determined by X-ray diffraction. The sample was mounted in the center of the sample holder on a glass slide and leveled to the correct height. The measurements were performed within a range in 2θ between 30° and 80° with a typical step size of 0.034° in 2θ. A position-sensitive detector, Lyn-Eye, was used to record diffraction data at a typical speed of 0.5 s/step which is equivalent to an effective time of 92 s/step for a scintillation counter. Characteristic functional groups involved in the synthesis were recorded using FTIR in the range of 400–4000 cm^−1^.

### 4.7. Stability of the Biosynthesized Silver Nanoparticles (OB-AgNPs)

To measure the stability of the OB-AgNPs, the method described by Elbagory et al. (2016) was followed [49]. Briefly, 100 µL of the OB-AgNPs solution was incubated with an equal volume (1:1) of the potato dextrose broth containing 1% DMSO. The stability of the OB-AgNPs was evaluated by measuring changes in the UV–Vis spectra after 24 h. Additionally, the stability of a sample stored at room temperature for two years was evaluated using UV-Vis, and DLS (ZP and average size distribution).

### 4.8. Fungal Strains and Culture Conditions

*Fusarium* strains used in this study were obtained from the Applied Microbial and Health Biotechnology Institute (AMHBI, Cape Peninsula University of Technology, Cape Town, South Africa) culture collection, including, *F. verticillioides* MRC 826-E, MRC 826-J and MRC 8559; *F. subglutinans* MRC 8553 and MRC 8554; *F. graminearum* MRC 6010; *F. proliferatum* MRC 8549 and MRC 8550; *F. globosum* MRC 6647 and MRC 6122 [35]. Freeze-dried spore suspensions of the different MRC strains were rehydrated using 1.0 mL dH_2_O and inoculated onto potato dextrose agar (PDA) plates and incubated at 26 °C for 2 weeks. For sporulation, Armstrong medium was inoculated with the different *Fusarium* strains from the PDA plates in Erlenmeyer flasks (250 mL) and incubated in a New Brunswick shaker incubator (Eppendorf, Hamburg, Germany) at 100 rpm and 26 °C for 5 days. Mycelial growth was removed by filtration of the suspensions through a double layer of sterile cheesecloth. Conidial suspensions were transferred to 50 mL sterile Falcon tubes and centrifuged at 4000 rpm for 10 min, and the supernatant was removed. Conidia were washed twice with sterile deionized water of volumes equivalent to that of the original suspension. Conidia were counted using a Neubauer hemocytometer, diluted to 1 × 10^6^ conidia/mL, and stored at 4 °C. Conidial viability (200 mL) was determined utilizing 1 mL Hoechst 33342 and 2 mL propidium iodide stain. The reaction mixture was incubated for 10 min at 22 ± 3 °C, and 100 mL of the stained samples was pipetted into an 8-well chamber slide (Thermo Scientific, Waltham, MA, USA). Samples were viewed using a 20× lens objective (LD A-plan 20×/0.35 Ph2), and images were taken using the Zeiss Axiocam 503 mono camera (Oberkochen, Germany), with the LED-Module/ex 511 nm/bandpass filter 555/25 for propidium iodide and the LED-Module/ex 385 nm/425/30 bandpass filter for Hoechst 33342. ZEN 2.6 (blue edition) software was used to acquire and process the images. 

### 4.9. Evaluation of Antifungal Inhibition of the O. basilicum Extract and Nanoparticles on ATP Production

The effect of the *O. basilicum* extract and their respective nanoparticles on conidial viability was evaluated using the BacTiter-Glo cell viability kit to measure ATP production. The concentration range (50–400 ng/µL) was selected to enable a direct comparison between the antifungal activity of the aqueous *O. basilicum* extract and the corresponding biosynthesized OB-AgNPs across identical treatment concentrations. The study was therefore designed to evaluate the enhancement in antifungal activity following nanoparticle synthesis rather than to determine the minimum inhibitory concentration of the crude extract. Briefly, 2000 ng/µL and 1000 ng/µL of extract and nanoparticles were respectively dissolved in 1.0 mL PDB containing 1% (*v*/*v*) DMSO. Serial dilutions were prepared to obtain the final concentrations of 400, 200, 100 and 50 ng/µL, respectively. Fifty microliters of the extract and nanoparticles were dispensed into the respective 96-well plates (white Falcon opaque plates with a low-evaporation lid; 1060 LJ: Amsterdam, Netherlands) with 5 replicates maintained for each treatment. Fifty microliters of the conidial suspensions (density of 1 × 10^6^ conidial/mL) were added to each respective well. To compare the antifungal susceptibility, 50 µL of only broth medium and 50 µL of conidial suspension (density of 1 × 10^6^ conidial/mL) without extract nor nanoparticles served as negative controls. Benomyl (2500 ng/µL) was used as a positive control. Plates were sealed with parafilm and incubated for 96 h at 26 °C. Hundred microliters (100 µL) BacTiter-GloTM microbial cell viability reagent was added to each respective wells after incubation. Plates were shaken gently for 2 min and allowed to stand in the dark for an additional 2 min. ATP determination of viable fungi inhibition was measured using the Veritas 96 microplate luminometer (Turner BioSystems Inc., Sunnyvale, CA, USA). Luminescence data obtained from the BacTiter-Glo™ (Promega Corporation, Madison, WI, USA) assay were analyzed using GraphPad Prism 5.01 software (GraphPad Software, Inc., La Jolla, CA, USA). To assess potential interference of the *O. basilicum* extract and OB-AgNPs with the BacTiter-Glo™ luminescence assay, control wells containing the extract or OB-AgNPs at the respective test concentrations in PDB medium, but without fungal conidia, were prepared. Following incubation, BacTiter-Glo™ reagent was added according to the manufacturer’s instructions, and luminescence was measured under identical conditions. The luminescence values obtained from these control wells were comparable to the background signal, indicating that neither the plant extract nor the nanoparticles contributed directly to the ATP-dependent luminescent signal.

### 4.10. Scanning Electron Microscopy (SEM) Analysis

*Fusarium* conidia were treated according to the procedure described before [35]. The lowest concentration (50 ng/µL) of both the *O. basilicum* extract and OB-AgNPs was selected for SEM analysis. *Fusarium* conidia (1 × 10^6^/mL) in Difco PDB were treated with the 50 ng/µL of *O. basilicum* extract and OB-AgNPs, respectively, in a final volume of 100 μL and incubated for 96 h at 26 °C. Treated cells were then fixed for 4 h in 2% (*v*/*v*) glutaraldehyde and 4% (*v*/*v*) paraformaldehyde (PFA) in 0.1 M Na-cacodylate buffer (pH 7.3) at 4 °C. To prevent drying, the cells were fixed in situ in the same primary fixative, using a multiwall plate/quad-Petri-dish, and covered. Post-fixation the cells were washed twice in 0.1 M Na-cacodylate containing 1% (*v*/*v*) aqueous OsO_4_ (1 h at 4 °C), and 3 times in double distilled H_2_O, and then dehydrated in a series of graded (50% to 70% to 90% [*v*/*v*]) ethanol and chemically dried using hexamethyldisilazane (HMDS) prior to SEM analysis. Samples were mounted onto a 12 mm aluminum SEM stub, using double-sided carbon conductive tape, and coated with 50 to 100-Å Au/Pd using a sputter coater (Leica EM ACE200, Wetzlar, Germany) for 3 min. Then they were visualized and identified at different magnifications under a scanning electron microscope (Carl Zeiss Microscopy, Jena, Germany). Images were captured using Smart SEM v6.09 (Zeiss Electron Imaging) and Oxford AZtecLive 6.2 (Oxford Instruments, Oxfordshire, UK).

### 4.11. Statistical Analysis

All experiments were performed using 5 biological repeats. Data are presented as the mean ± standard deviation (SD). Statistical analyses were performed using GraphPad Prism version 5.01 (GraphPad Software Inc., La Jolla, CA, USA). Comparisons between treatment groups were performed using one-way ANOVA followed by Tukey’s multiple comparisons test. Differences were considered statistically significant at *p* < 0.05. Statistical significance is indicated as follows: ns, not significant (*p* ≥ 0.05); *p* < 0.05 (*); *p* < 0.01 (**); *p* < 0.001 (***); and *p* < 0.0001 (****).

## 5. Conclusions

The aqueous extract of *O. basilicum* contains bioactive secondary metabolites capable of mediating the synthesis and stabilization of AgNPs. The synthesized nanoparticles were characterized using conventional analytical methods and demonstrated stability in up to two years. The study evaluated the efficacy of *O. basilicum* aqueous extract and the biosynthesized OB-AgNPs against selected *Fusarium* species known for their mycotoxin-producing capabilities. The highest tested concentration (400 ng/µL) of the aqueous extract exhibited antifungal activity only against *F. globosum* MRC 6122. In contrast, OB-AgNPs demonstrated near-complete fungicidal activity against most strains, except for *F. proliferatum* MRC 8549 where it had no effect. The antifungal activity of the extract may be attributed to the combined effect of multiple polyphenolic and other bioactive compounds present in the plant material. Overall, the results demonstrate that OB-AgNPs possess significantly enhanced antifungal activity compared to crude extract and exhibit strong potential for controlling *Fusarium*-related diseases. The combination of biological synthesis, high efficacy at low concentrations, and pronounced structural disruption of fungal cells suggests that OB-AgNPs may serve as effective and environmentally compatible alternatives to conventional fungicides. Future studies should focus on in vivo evaluations, formulation development, and safety assessments to facilitate the translation of these findings into practical agricultural applications.

## Figures and Tables

**Table 1:** Ultraviolet–visible (UV-Vis) absorbance, zeta potential (ZP), polydispersity index (PDI), particle size measurement using dynamic light scattering (DLS), high-resolution transmission electron microscopy (HRTEM), and powder X-ray diffraction (PXRD) of the biosynthesized silver nanoparticles (OB-AgNPs).

Sample	UV-Vis λ_max_ (nm)	ZP (mV)	PDI	Average Hydrodynamic Size (nm) *	TEM Size Range (nm)	Average Size/XRD ** (nm)
OB-AgNPs	437 ± 2	–16.3 ± 7.9	0.361	57.08 *	18.5–37.4	~11.18

* The size distribution by intensity was bimodal. Small nanoparticles with an average diameter of 5.2 ± 0.7 nm and larger nanoparticles with an average diameter distribution of 67.8 ± 20 nm can be observed in Figure 3. ** Calculated using the Debye–Scherrer equation.

## Data Availability

Raw data and data supporting the results can be requested from https://www.cput.ac.za/lib, upon request.

## Supplementary Materials

The following supporting information can be downloaded at https://www.mdpi.com/article/10.3390/molecules31172985/s1: Figure S1: Visual comparison of the *Ocimum basilicum* aqueous extract and the biosynthesized silver nanoparticles; Figure S2: Liquid chromatography–mass spectrometry (LC-MS) chromatogram of the aqueous extract from *Ocimum basilicum*; Table S1: Phytochemicals identified in the aqueous extract of *Ocimum basilicum*; Figure S3: High-resolution transmission electron microscopy (HRTEM) image of the biosynthesized silver nanoparticles (OB-AgNPs); Figure S4: X-ray diffraction (XRD) pattern of biosynthesized silver nanoparticles (OB-AgNPs); Figure S5: Zeta potential distribution and hydrodynamic size distribution of the biosynthesized silver nanoparticles (OB-AgNPs) after 2 years; Table S2: Conidial viability parameters expressed as % ATP (adenosine triphosphate) production compared to the control after 96 h of exposure to *Ocimum basilicum* extract and the biosynthesized silver nanoparticles (OB-AgNPs).

## References

[1] Ekwomadu T.I., Mwanza M. (2023). *Fusarium* Fungi Pathogens, Identification, Adverse Effects, Disease Management, and Global Food Security: A Review of the Latest Research. Agriculture.

[2] Hof H., Schrecker J. (2024). *Fusarium* spp.: Infections and Intoxications. GMS Infect. Dis..

[3] IARC (2002). Some Traditional Herbal Medicines, Some Mycotoxins, Naphthalene and Styrene. IARC Monographs on the Evaluation of Carcinogenic Risks to Humans.

[4] Domijan A.-M. (2012). Fumonisin B1: A Neurotoxic Mycotoxin/Fumonizin B1: Neurotoksični Mikotoksin. Arch. Ind. Hyg. Toxicol..

[5] Mwangi R.W., Mustafa M., Charles K., Wagara I.W., Kappel N. (2023). Selected Emerging and Reemerging Plant Pathogens Affecting the Food Basket: A Threat to Food Security. J. Agric. Food Res..

[6] Madhusudanan M., Zhang J., Pandit S., Singh P., Jeong G., Khan F., Mijakovic I. (2025). Green Synthesis of Silver Nanoparticles: A Review of Polymer and Antimicrobial Drug Combinations for Enhanced Antimicrobial Applications. Adv. NanoBiomed Res..

[7] Dheyab M.A., Aziz A.A., Nowfal S.H., Braim F.S., Abdullah W., Kasasbeh W.H., Jameel M.S., Alanezi S.T., Alrosan M., Oladzadabbasabadi N. (2025). Sustainable Green Synthesis of Silver Nanoparticles for Safer Biomedical Application. J. Environ. Chem. Eng..

[8] Shahzadi S., Fatima S., Ul Ain Q., Shafiq Z., Janjua M.R.S.A. (2025). A Review on Green Synthesis of Silver Nanoparticles (SNPs) Using Plant Extracts: A Multifaceted Approach in Photocatalysis, Environmental Remediation, and Biomedicine. RSC Adv..

[9] Selvam K., Ragu Prasath A., Alanazi A.K. (2026). A Review of Recent Developments in Green Synthesis of Silver Nanoparti-cles: Antioxidant and Antibacterial Applications. Microsc. Res. Tech..

[10] Eker F., Akdaşçi E., Duman H., Bechelany M., Karav S. (2025). Green Synthesis of Silver Nanoparticles Using Plant Extracts: A Comprehensive Review of Physicochemical Properties and Multifunctional Applications. Int. J. Mol. Sci..

[11] Zehra S.H., Ramzan K., Viskelis J., Viskelis P., Balciunaitiene A. (2025). Advancements in Green Synthesis of Silver-Based Nanoparticles: Antimicrobial and Antifungal Properties in Various Films. Nanomaterials.

[12] Mohsen A., Abdel-Goad M.A. (2025). Silver Nanoparticles from Nature: Green Synthesis Methods and Applications—A Review. J. Adv. Eng. Trends.

[13] Tudi M., Daniel Ruan H., Wang L., Lyu J., Sadler R., Connell D., Chu C., Phung D.T. (2021). Agriculture Development, Pesticide Application and Its Impact on the Environment. Int. J. Environ. Res. Public Health.

[14] Lahlali R., Ezrari S., Radouane N., Kenfaoui J., Esmaeel Q., El Hamss H., Belabess Z., Barka E.A. (2022). Biological Control of Plant Pathogens: A Global Perspective. Microorganisms.

[15] Al-Audah S.A., Alghamdi A.I., Alsanie S.I., Alabdalla N.M., Alawdah A., Alenezi N., AlShammari A., Taha I., Albarrag A., Aldakeel S. (2026). Green Synthesis of Silver Nanoparticles with Antibacterial, Anti-Inflammatory, and Antioxidant Activity Using *Convolvulus arvensis*. Int. J. Mol. Sci..

[16] Al-Agamy D.M., Atef A., Alqhtani A.H., Ali A., Yosri M. (2026). Investigation of the Impact of Myco-Synthesized Silver Nanoparticles against Soil-Borne *Fusarium oxysporum*: Ultrastructure Appraisal. Pol. J. Environ. Stud..

[17] Nasr Azadani F., Madani M., Karimi J., Sepahvand S. (2024). Green synthesis of silver nanoparticles by *Fusarium oxysporum* and its function against *Aspergillus* and *Fusarium* fungi. Indian J. Microbiol..

[18] Shahrajabian M.H., Sun W., Cheng Q. (2020). Chemical Components and Pharmacological Benefits of Basil (*Ocimum basilicum*): A Review. Int. J. Food Prop..

[19] Kousar M.U., Jabeen A., Malik M.A., Hussain S.Z., Manzoor S., Mukhtar T., Yousuf M., Mushtaq A., Yaseen M. (2026). Basil *Ocimum basilicum* as a Bioactive Resource: Chemical Composition and Extraction Innovation. Food Humanit..

[20] Handayani D.P., Mahfud M., Kusuma H.S. (2025). Extraction Technologies and Bioactive Applications of *Ocimum basilicum*: A Bibliometric and Systematic Review. Sustain. Chem. Clim. Action..

[21] Zhakipbekov K., Turgumbayeva A., Akhelova S., Bekmuratova K., Blinova O., Utegenova G., Shertaeva K., Sadykov N., Tastambek K., Saginbazarova A. (2024). Antimicrobial and Other Pharmacological Properties of *Ocimum basilicum*, Lamiaceae. Molecules.

[22] Huang X., Devi S., Bordiga M., Brennan C.S., Xu B. (2023). Phenolic Compounds Mediated Biosynthesis of Gold Nanoparticles and Evaluation of Their Bioactivities: A Review. Int. J. Food Sci. Technol..

[23] Sidhu A.K., Verma N., Kaushal P. (2022). Role of Biogenic Capping Agents in the Synthesis of Metallic Nanoparticles and Evaluation of Their Therapeutic Potential. Front. Nanotechnol..

[24] Figat A.M., Bartosewicz B., Liszewska M., Budner B., Norek M., Jankiewicz B.J. (2023). α-Amino Acids as Reducing and Capping Agents in Gold Nanoparticles Synthesis Using the Turkevich Method. Langmuir.

[25] Al-Mutaani J., Zorgui T., Mohamed A.A., Smetanska I., Zourgui L., Missaoui N. (2025). Comparative Antioxidant Activity of Four Omani Medicinal Plants: *Ocimum basilicum*, *Teucrium polium*, *Caralluma arabica*, and *Cleome amblyocarpa*. Open Vet. J..

[26] Srivastava V., Joseph A.M., Kumar P., Ansari K., Trivedi K., Parmar N., Patel B., Patel A., Sahoo D.K., Jhala D. (2025). Comparative Metabolomic and Pharmacognostic Analysis of *Ocimum basilicum* L. Extracts: Insights into Antioxidant, Toxicity, and Anticancer Potentials. Int. J. Food Sci..

[27] Zafar N., Rafique A., Naz S., Nazir M.M., Ashraf A. (2025). Unveiling the in Vitro, in Vivo Anti-Inflammatory Potential of *Ocimum basilicum* and in Silico Analysis of Its Phytocompounds Targeting COXs Proteins. J. Pharm. Pharmacol..

[28] Abdullah H.T., Al-Bayati A.M., Saleh A.N.A., AlSalihi K.A., Abed H.H. (2025). Effect of *Ocimum basilicum* Herbs Extract on Pro-Inflammatory Cytokines in Ethanol-Induced Liver Damage in Rats. Wiad. Lek..

[29] Teofilović B., Tomas A., Martić N., Stilinović N., Čapo I., Grujić-Letić N., Gligorić E., Rašković A. (2025). Therapeutic Potential of Basil *Ocimum basilicum* L. Aqueous Extract: Impact on Glycemia and Oxidative Stress in Normoglycemic and Diabetic Rats. Pharmazie.

[30] Al-Khazaali I.T.H., Zeiny S.S.M. (2025). Impact of Nanoparticle *Ocimum basilicum* on Lipid Profile in Diabetes Mellitus Albino Rats. Kufa J. Vet. Med. Sci..

[31] Jameel A., Malik H.R. (2025). GC-MS Assay to Aqueous and Alcoholic Extracts of *Ocimum basilicum* L. and Detection Antibacterial Activity. J. Med. Plants By-Prod..

[32] Rani U., Khan T.H., Riaz M.U., Saeed A., Tooheed B., Hayat S. (2025). Comparative Phytochemical Profiling and Antimicrobial Activity of Different Plant Parts of *Ocimum basilicum* L.. Glob. For..

[33] Ababutain I. (2019). Antimicrobial Activity and Gas Chromatography-Mass Spectrometry (GC-MS) Analysis of Saudi Arabian *Ocimum basilicum* Leaves Extracts. J. Pure Appl. Microbiol..

[34] Fatima H., Hamdani S.D.A., Jadoon L., Gul A., Rajput T.A., Munir F., Amir R., Babar M.M. (2026). Effective Attenuation of Multidrug Resistant *Staphylococcus aureus* by Hydroponically Grown Basil Synthesized Nanoparticles in Burn Wound Model. Bio Nanosci..

[35] Omoruyi B.E., Volschenk H., Gelderblom W.C.A., Lilly M. (2023). Biocontrol of *Fusarium* Species Utilizing Indigenous Rooibos and Honeybush Extracts. Microbiol. Spectr..

[36] Chulze S.N., Tittlemier S., Torres A.M. (2023). Editorial: *Fusarium* Species as Plant and Human Pathogens, Mycotoxin Producers, and Biotechnological Importance. Front. Fungal Biol..

[37] Mossa M.I., El-Sharkawy E.E., ElSharawy A.A. (2023). Antifungal Activity of Silver Nanoparticles Green Biosynthesis from the Extract of *Zygophyllum album* (L.f.) on *Fusarium* Wilt. J. Plant Prot. Res..

[38] Feksa H.R., Couto H.T.Z., Garozi R., Almeida J.L., Gardiano C.G., Tessmann D.J. (2019). Pre- and Postinfection Application of Strobilurin-Triazole Premixes and Single Fungicides for Control of *Fusarium* Head Blight and Deoxynivalenol Mycotoxin in Wheat. Crop Prot..

[39] Mwangi R., Kimani N.M. (2026). Natural Pesticides in Crop Protection: Comparative Advantages, Ecological Risks, and Future Directions. J. Chem..

[40] Sinang F.M., Mahmud Ab Rashid N.K., Ismail S.I., Masdor N.A., Samsudin N.I.P. (2026). Biological Control against Mycotoxigenic Fungi and Mycotoxins: Formulations, Challenges, and Future Prospects. J. Appl. Microbiol..

[41] Kalimuthua N., Subashchandrabose S., Meganathan C. (2026). Dual-Extract Driven AgCl Nanoparticles (AgCl-NP): Mechanistic Insights into Bioactivity, Ecotoxicity, and Targeted Molecular Interactions. Appl. Nanosci..

[42] Tabassum R.Z., Mehmood A., Khalid A.U.R., Ahmad K.S., Khan M.A.R., Amjad M.S., Raffi M., Khan G.-E.-L., Mustafa A. (2024). Green Synthesis of Silver Nanoparticles for Antifungal Activity against Tomato *Fusarium* Wilt Caused by *Fusarium oxysporum*. Biocatal. Agric. Biotechnol..

[43] Darwazah R., Salem N., Awwad A. (2026). Green-Synthesized Silver Nanoparticles as Antifungal Agents against Tomato Vascular Wilt. Int. J. Agric. Biosci..

[44] Malapermal V., Botha I., Krishna S.B.N., Mbatha J.N. (2017). Enhancing Antidiabetic and Antimicrobial Performance of *Ocimum basilicum* and *Ocimum sanctum* (L.) Using Silver Nanoparticles. Saudi J. Biol. Sci..

[45] Ali S.A., Osman M.E., Mohamed E.T. (2025). Eco-Friendly Biosynthesis of Silver Nanoparticles Using Marine-Derived *Fusarium equiseti*: Optimization, Characterization, and Evaluation of Antimicrobial, Antioxidant, and Cytotoxic Activities. World J. Microbiol. Biotechnol..

[46] Boualares I., Derouiche S., Niemann J. (2024). HPLC-Q-TOF-MS Analysis of Phenolic Compounds, in Vitro Biological Activities and in Vivo Acute Toxicity Evaluation of *Ocimum basilicum* L.. Fresenius Environ. Bull..

[47] Dev Sharma A., Kaur I., Angish S., Thakur A., Sania S., Singh A. (2022). Comparative Phytochemistry, Antioxidant, Anti-diabetic, and Anti-Inflammatory Activities of Traditionally Used *Ocimum basilicum* L. *Ocimum gratissimum* L., and *Ocimum tenuiflorum* L. Bio Technol..

[48] Deepa M.K., Suryaprakash T.N.K., Kumar P. (2017). Eco friendly green synthesized silver nanoparticle with *Ocimum basilicum* leaves aqueous extract. J. Innov. Pharm. Biol. Sci..

[49] Elbagory A., Cupido C., Meyer M., Hussein A. (2016). Large Scale Screening of Southern African Plant Extracts for the Green Synthesis of Gold Nanoparticles Using Microtitre-Plate Method. Molecules.

[50] Hernández-Pinero J.L., Terrón-Rebolledo M., Foroughbakhch R., Moreno-Limón S., Melendrez M.F., Solís-Pomar F., Pérez-Tijerina E. (2016). Effect of Heating Rate and Plant Species on the Size and Uniformity of Silver Nanoparticles Synthesized Using Aromatic Plant Extracts. Appl. Nanosci..

[51] Kiani H.S., Ali B., Al-Sadoon M.K., Al-Otaibi H.S., Ali A. (2023). LC-MS/MS and GC-MS Identification of Metabolites from the Selected Herbs and Spices, Their Antioxidant, Anti-Diabetic Potential, and Chemometric Analysis. Processes.

[52] Farag M.A., Ezzat S.M., Salama M.M., Tadros M.G. (2016). Anti-Acetylcholinesterase Potential and Metabolome Classifica-tion of 4 *Ocimum* Species as Determined via UPLC/qTOF/MS and Chemometric Tools. J. Pharm. Biomed. Anal..

[53] Wójciak M., Paduch R., Drozdowski P., Żuk M., Wójciak W., Tyszczuk-Rotko K., Feldo M., Sowa I. (2024). Ultra-Performance Liquid Chromatography and Mass Spectrometry Characterization, and Antioxidant, Protective, and Anti-Inflammatory Activity, of the Polyphenolic Fraction from *Ocimum basilicum*. Molecules.

[54] Nadeem H.R., Akhtar S., Sestili P., Ismail T., Neugart S. (2022). Toxicity, Antioxidant Activity, and Phytochemicals of Basil (*Ocimum basilicum* L.) Leaves Cultivated in Southern Punjab, Pakistan. Foods.

[55] Vidaković V., Vujić B., Jadranin M., Novaković I., Trifunović S., Tešević V., Mandić B. (2024). Qualitative Profiling, Antioxidant and Antimicrobial Activities of Polar and Nonpolar Basil Extracts. Foods.

[56] Abdel-razakh H.H., Bakari G.G., Park J.-S., Pan C.-H., Hoza A.S. (2024). Phenolic Contents and Antioxidant Properties of *Bauhinia rufescens*, *Ocimum basilicum* and *Salvadora persica*, Used as Medicinal Plants in Chad. Molecules.

[57] Mahmoud E., Starowicz M., Ciska E., Topolska J., Farouk A. (2022). Determination of Volatiles, Antioxidant Activity, and Polyphenol Content in the Postharvest Waste of *Ocimum basilicum* L.. Food Chem..

[58] Cruz-Luna A.-R., Gutiérrez M.C., Karaaslan M.A., Soto-Castro D., Vásquez-López A. (2026). Green Synthesis of Biogenic Silver Nanoparticles from *Agave potatorum* Leaves and Their Potent Antifungal Activity against *Neocosmospora* sp. and *Fusarium nirenbergiae*. Biocatal. Agric. Biotechnol..

[59] Ghasemi M., Govahi M., Litkohi H.R. (2025). Green Synthesis of Silver Nanoparticles (AgNPs) and Chitosan-Coated Silver Nanoparticles (CS-AgNPs) Using *Ferula gummosa* Boiss. Gum Extract: A Green Nano Drug for Potential Applications in Medicine. Int. J. Biol. Macromol..

[60] Sharma S., Bose A., Biswas S., Sen S., Roy I. (2025). *Cyperus rotundus* Mediated Green Synthesis of Silver Nanoparticles for Antibacterial Wound Dressing Applications. Sci. Rep..

[61] Yusuf-Salihu B.O., Abdulmumini S.A., Bajepade T.T., Durosinmi H.A., Kazeem M.O., Ajayi V.A., Lateef A. (2025). Novel Green Synthesis of Silver Nanoparticles from Empty Fruit Bunch Waste: Biomedical Applications and Mechanistic Insights. Next Nanotechnol..

[62] Ashraf H., Anjum T., Ahmad I.S., Ahmed R., Aftab Z.-H., Rizwana H. (2025). Phytofabricated Silver Nanoparticles Unlock New Potential in Tomato Plants by Combating Wilt Infection and Enhancing Plant Growth. Sci. Rep..

[63] Deore S.L., Ingole S.R., Baviskar B.A., Kide A.A. (2020). Comparative Pharmacognostical, Phytochemical and Biological Evaluation of Five *Ocimum* Species. Pharmacogn. J..

[64] Wu Q., Cen F., Xie Y., Ning X., Wang J., Lin Z., Huang J. (2025). Nanoparticle-Based Antifungal Therapies: Innovations, Mechanisms and Future Prospects. PeerJ.

[65] Nagaraj S., Manivannan S., Narayan S. (2021). Potent Antifungal Agents and Use of Nanocarriers to Improve Delivery to the Infected Site: A Systematic Review. J. Basic. Microbiol..

[66] Bouqellah N.A., Abdulmajeed A.M., Alharbi F.K.R., Mattar E., Al-Sarraj F., Abdulfattah A.M., Hassan M.M., Baazeem A., Al-Harthi H.F., Musa A. (2025). Optimizing Encapsulation of Garlic and Cinnamon Essential Oils in Silver Nanoparticles for Enhanced Antifungal Activity against *Botrytis cinerea* Pathogenic Disease. Physiol. Mol. Plant Pathol..

[67] Dube E., Okuthe G.E. (2025). Silver Nanoparticle-Based Antimicrobial Coatings: Sustainable Strategies for Microbial Contamination Control. Microbiol. Res..

[68] Alabrahim O.A.A., Abdeldayem A.M., Azzazy H.M.E.-S. (2025). Green Synthesis of Metallic Nanoparticles Using *Pistacia* Species: Improved Stability and Biological Activities. Nanoscale Adv..

[69] Applebach E., Amburgey-Crovetti K., Bakotic W.L., Adhikari T.B., Wiederhold N.P., Hodges S., Steen T.Y., Calderone R., Li D. (2025). Prevalence and diversity of antifungal resistance in *Fusarium* isolates across clinical and agricultural settings in the United States. Antimicrob. Agents Chemother..

[70] Sivalingam A.M., Pandian A., Kedari G.S.R., Kumar V. (2025). Identification of Bio-Active Compounds and Biochemical Characterization of Green Synthesized Silver Nanoparticles (AgNPs) against Selected Uropathogenic Antimicrobial Activity. J. Inorg. Organomet. Polym. Mater..

[71] Pradhan U., Prajapati J.P., Majhi P., Sahu D., Singh R.K., Mallick S., Shukla A.K. (2025). Green Synthesis and Characterization of *Blumea sinuata* Silver Nanoparticles: Antibacterial, Antifungal, and Antioxidant Properties. Nanoscale Adv..

[72] Abd-Elhamed W., Mohamed A.A., Saad Z.H., Hassanien S.E.-S.I., Salem M.Z.M., El-Hefny M. (2025). Green synthesis of Silver Nanoparticles Mediated By *Solanum nigrum* Leaf Extract and Their Antifungal Activity Against Pine Pathogens. Sci. Rep..

[73] Mohany M., Ali J., Wahab A., Fozia F., Shah S.M., Gul R., Gul A., Ahmad I., Milošević M., Al-Rejaie S.S. (2025). Green Synthesized AgNPs of the *Anchusa arvensis* Aqueous Extract Resulting in Impressive Protein Kinase, Antioxidant, Antibacterial, and Antifungal Activities. Z. Für Naturforsch..

[74] Kumar M., Saini R.V., Gupta M., Singh R. (2025). Green Synthesis of Silver Nanoparticle (Cha-AgNPs) Using *Chenopodium album* Extract and Evaluation of Their Antifungal Potential against Pathogenic Fungi. Biomass Convers. Biorefinery.

[75] Wang J., Xie T., Zhang S., Shao H., Wu J., Liu X., Wu Q., Wang Y., He R. (2025). Green Synthesis of Silver Nanozymes for Synergistic Antifungal Application on Crops. ACS Appl. Nano Mater..

[76] Qureshi A.K., Farooq U., Shakeel Q., Ali S., Ashiq S., Shahzad S., Tariq M., Seleiman M.F., Jamal A., Saeed M.F. (2023). The Green Synthesis of Silver Nanoparticles from *Avena fatua* Extract: Antifungal Activity against *Fusarium oxysporum* f.sp. Lycopersici. Pathogens.

