# Supplementary material for: Biosynthesis of Silver Nanoparticles Using Ocimum basilicum Extract and Evaluation of Their Antifungal Efficacy Against Selected Fusarium Plant Pathogens
Source: Molecules. 2026 Aug 26;31(17):2985. doi: 10.3390/molecules31172985 (PMC13567591; doi:10.3390/molecules31172985)
Supplement: Supplementary file 1 [file molecules-31-02985-s001.zip › molecules-4355136-supplementary.pdf]

molecules

Supplementary Materials

Biosynthesis of silver nanoparticles (AgNPs) using *Ocimum basilicum* extract and evaluation of their antifungal efficacy against selected *Fusarium* plant pathogens.

Ali O.E. Eltahir, Beauty E. Omoruyi, Mariska Lilly, Robert C Luckay, and Ahmed A. Hussein

Supplementary information

Figure S1. Visual comparison of the *Ocimum basilicum* aqueous extract and the biosynthesized silver nanoparticles.
Figure S2. Liquid chromatography-mass spectrometry (LC-MS) chromatogram of the aqueous extract from *Ocimum basilicum.*
Table S1. Phytochemicals identified in the aqueous extract of *Ocimum basilicum.*
Figure S3: High resolution transmission electron microscopy (HRTEM) image of the biosynthesized silver nanoparticles (OB-AgNPs).
Figure S4: X-ray diffraction (XRD) pattern of biosynthesized silver nanoparticles (OB-AgNPs).
Figure S5. Zeta potential distribution and hydrodynamic size distribution of the biosynthesized silver nanoparticles (OB-AgNPs) after 2 year.
Table S2. Conidial viability parameters expressed as % ATP (Adenosine triphosphate) production compared to the control after 96 h of exposure to Ocimum basilicum extract and the biosynthesized silver nanoparticles (OB-AgNPs).

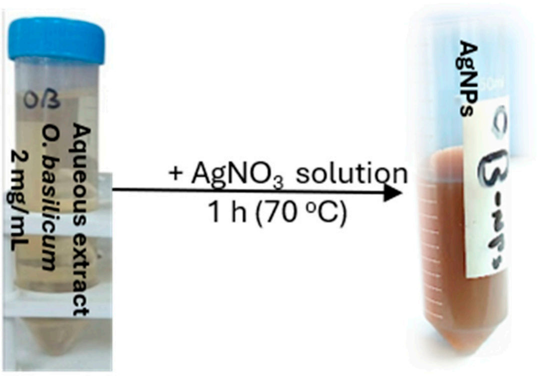

Figure S1. Visual comparison of the *Ocimum basilicum* aqueous extract and the biosynthesized silver nanoparticles.

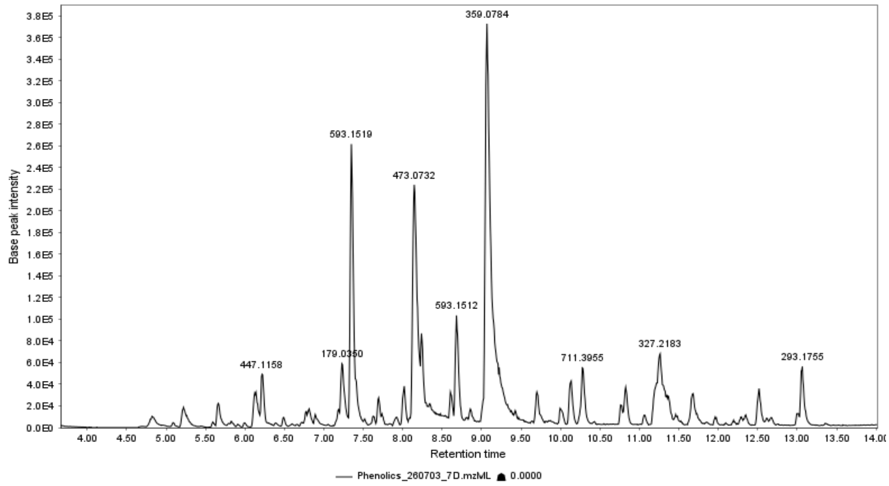

Figure S2. Liquid chromatography-mass spectrometry (LC-MS; ESI-negative mode) chromatogram of the aqueous extract from *Ocimum basilicum.*

Table S1. Phytochemicals identified in the aqueous extract of *Ocimum basilicum.*

Compound Name	Rt (min)	Molecular Weight (g/mol)	Final Concentration (μg / mg extract)*
Syringic acid	5.662	197.0461	3.86±0.2
Caftaric acid	6.132	311.0411	5.88±0.5
Aralidioside	6.224	447.1154	8.28±1.6
Caffeic acid	7.236	179.0355	10.06±1.3
Vicenin-2	7.355	593.1521	42.04±6.3
Chicoric acid	8.145	473.0732	39.64±8.3
Rutin	8.237	609.1460	14.38±3.0
Sagerinic acid	8.611	719.1626	5.46±0.4
Nicotiflorin	8.681	593.1512	16.88±0.9
Rosmarinic acid	9.075	360.3180	65.00±2.2

*Calculated from duplicate measurements.

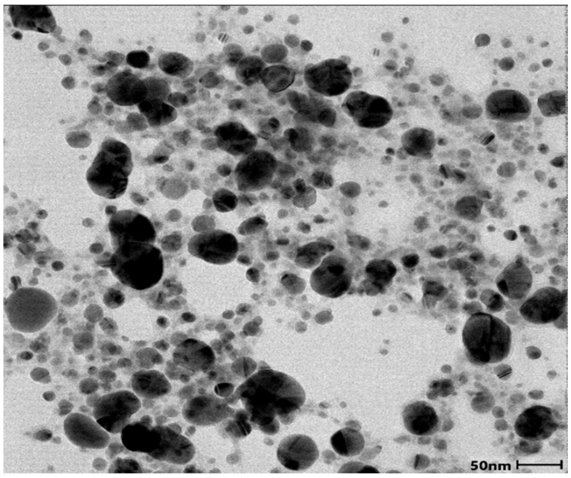

Figure S3: High resolution transmission electron microscopy (HRTEM) image of the biosynthesized silver nanoparticles (OB-AgNPs).

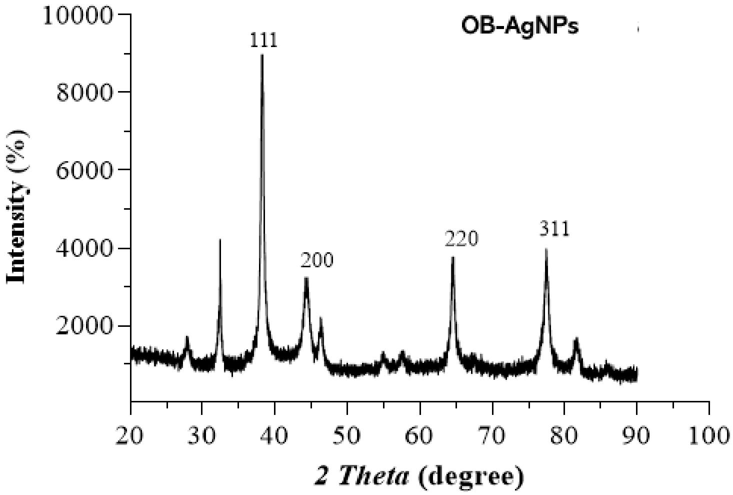

Figure S4. X-ray diffraction (XRD) pattern of the biosynthesized silver nanoparticles (OB-AgNPs).

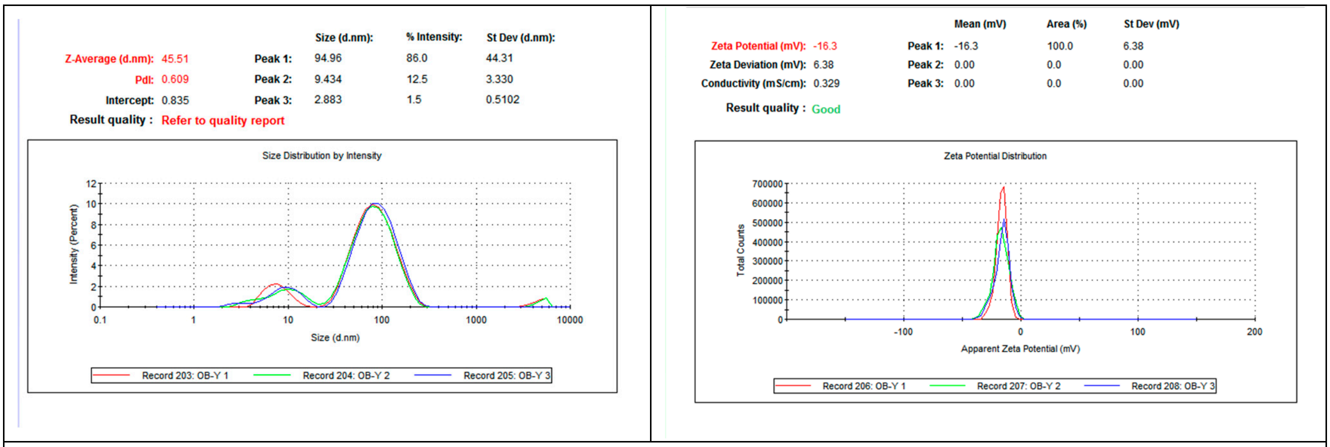

Figure S5. Zeta potential distribution and hydrodynamic size distribution of the biosynthesized silver nanoparticles (OB-AgNPs) after 2 years

Table S2. Conidial viability parameters expressed as % ATP (Adenosine triphosphate) production compared to the control after 96 h of exposure to *Ocimum basilicum* extract and the biosynthesized silver nanoparticles (OB-AgNPs).

Strain	Control	*Ocimum basilicum* extract concentration (ng/µL)				Biosynthesized silver nanoparticles (OB-AgNPs) concentration (ng/µL)				Benomyl concentration (ng/µL)
		400	200	100	50	400	200	100	50	2500
Fusarium verticillioides										
**MRC 826-E**	100 ± 3.43	93 ± 0.83	100 ± 1.18	105 ± 1.50	109 ± 1.99	0.54 ± 0.03	0.69 ± 0.09	1.15 ± 0.03	20.3 ± 1.74	61.3 ± 0.65
**MRC 826-J**	100 ± 2.46	98 ± 0.45	102 ± 4.13	118 ± 5.47	134 ± 5.33	0.56 ± 0.01	1.72 ± 0.17	4.73 ± 0.32	59.9 ± 2.56	78.9 ± 2.37
**MRC 8559**	100 ± 1.78	258 ±12.60	290 ± 5.72	330 ± 30.30	366 ± 37.40	3.08 ± 0.16	4.26 ± 0.22	4.50 ± 0.30	68.3 ± 0.63	90.3 ± 2.36
Fusarium proliferatum										
**MRC 8550**	100 ± 0.88	101 ±0.93	101 ± 0.61	102 ± 1.08	103 ± 0.87	70.1 ± 2.36	72.9 ± 1.79	80.6 ± 0.66	91.8 ± 2.82	10.7 ± 0.42
**MRC 8549**	100 ± 5.51	98 ± 4.58	109 ± 1.45	141 ± 5.85	144 ± 19.00	82.3 ± 0.16	95.8 ± 10.4	93.6 ± 0.53	105.7 ± 0.17	72.8 ± 1.29
Fusarium subglutinans										
**MRC 8553**	100 ± 5.40	95 ± 2.08	109 ± 3.30	124 ± 3.60	155 ± 1.79	2.52 ± 0.09	2.77 ± 0.13	4.38 ± 0.13	5.91 ± 0.25	80.7 ± 0.08
**MRC 8554**	100 ± 2.74	114 ±1.85	126 ± 2.57	163 ± 2.34	171 ± 4.25	2.43 ± 0.27	4.18 ± 0.15	8.35 ± 0.16	9.01 ± 0.22	14.7 ± 1.02
Fusarium graminearum										
**MRC 6010**	100 ± 2.65	131 ±4.90	140 ± 1.91	154 ± 2.81	162 ± 3.60	0.07 ± 0.01	0.09 ± 0.01	0.14 ± 0.00	0.22 ± 0.01	0.50 ± 0.05
Fusarium globosum										
**MRC 6647**	100 ± 6.53	156 ±11.00	169 ± 6.60	198 ± 5.30	198 ± 8.53	2.57 ± 0.12	5.67 ± 0.68	35.9 ± 2.33	49.3 ± 2.01	32.7 ± 4.75
**MRC 6122**	100 ± 5.26	66 ± 7.27	70 ± 8.95	71 ± 2.50	87 ± 1.26	4.83 ± 0.24	4.95 ± 0.32	5.19 ± 0.14	6.19 ± 0.27	2.74 ± 0.16

Values represent means ± standard deviations of five replications of at least two experiments. Abbreviations: ATP- adenosine triphosphate; % ATP production – Percentage spore viability of cell energy production
